# Drug Loaded 3D-Printed Poly(ε-Caprolactone) Scaffolds for Local Antibacterial or Anti-Inflammatory Treatment in Bone Regeneration

**DOI:** 10.3390/polym15193957

**Published:** 2023-09-30

**Authors:** Mariia Stepanova, Ilia Averianov, Iosif Gofman, Natalia Shevchenko, Artem Rubinstein, Tatiana Egorova, Andrey Trulioff, Yulia Nashchekina, Igor Kudryavtsev, Elena Demyanova, Evgenia Korzhikova-Vlakh, Viktor Korzhikov-Vlakh

**Affiliations:** 1 Institute of Macromolecular Compounds, Russian Academy of Sciences, 199004 St. Petersburg, Russia; maristepanova@hq.macro.ru (M.S.); averianovilia@gmail.com (I.A.); gofman@imc.macro.ru (I.G.); natali.shevchenko29@gmail.com (N.S.); vlakh@hq.macro.ru (E.K.-V.); 2 Institute of Experimental Medicine, 197376 St. Petersburg, Russia; arrubin6@mail.ru (A.R.); trulioff@gmail.com (A.T.); igorek1981@yandex.ru (I.K.); 3 State Research Institute of Highly Pure Biopreparations FMBA of Russia, 197110 St. Petersburg, Russia; egorova25tat@yandex.ru (T.E.); lenna_22@mail.ru (E.D.); 4 Institute of Cytology, Russian Academy of Sciences, 194064 St. Petersburg, Russia; ulychka@mail.ru; 5 School of Biomedicine, Far Eastern Federal University, 10 Ajax Bay, Russky Island, 690922 Vladivostok, Russia; 6 Institute of Chemistry, Saint-Petersburg State University, 198504 St. Petersburg, Russia

**Keywords:** 3D-printed polymer materials, scaffolds, implantable drug delivery systems, ciprofloxacin, dexamethasone, drug release, antimicrobial activity, anti-inflammatory effect

## Abstract

Annual bone grafting surgeries due to bone fractures, resections of affected bones, skeletal anomalies, osteoporosis, etc. exceed two million worldwide. In this regard, the creation of new materials for bone tissue repair is one of the urgent tasks of modern medicine. Additive manufacturing, or 3D printing, offers great opportunities for the development of materials with diverse properties and designs. In this study, the one-pot technique for the production of 3D scaffolds based on poly(ε-caprolactone) (PCL) loaded with an antibiotic or anti-inflammatory drug was proposed. In contrast to previously described methods to prepare drug-containing scaffolds, drug-loaded PCL scaffolds were prepared by direct 3D printing from a polymer/drug blend. An investigation of the mechanical properties of 3D-printed scaffolds containing 0.5–5 wt% ciprofloxacin (CIP) or dexamethasone (DEX) showed almost no effect of the drug (compression modulus ~70–90 MPa) compared to unfilled PCL (74 MPa). At the same time, introducing the drug and increasing its content in the PCL matrix contributed to a 1.8–6.8-fold decrease in the specific surface area of the scaffold, depending on composition. The release of CIP and DEX in phosphate buffer solution and in the same buffer containing lipase revealed a faster release in enzyme-containing medium within 45 days. Furthermore, drug release was more intensive from scaffolds with a low drug load. Analysis of the release profiles using a number of mathematical dissolution models led to the conclusion that diffusion dominates over other probable factors. In vitro biological evaluation of the scaffolds containing DEX showed moderate toxicity against osteoblast-like and leukemia monocytic cells. Being 3D-printed together with PCL both drugs retain their biological activity. PCL/CIP and PCL/DEX scaffolds demonstrated antibacterial properties against *Pseudomonas aeruginosa* (a total inhibition after 48 h) and anti-inflammatory activity in experiments on TNFα-activated monocyte cells (a 4-time reduction in CD-54 expression relative to control), respectively.

## 1. Introduction

Annual bone grafting surgeries due to bone fractures, resections of affected bones, skeletal anomalies, osteoporosis, etc. exceed two million worldwide [1]. Autografting is the gold standard for bone regeneration because the autologous bone is a non-immunogenic, osteoinductive and osteoconductive biomaterial. However, the use of autografts has several disadvantages such as limited availability, variable quality, and increased operative time [1]. In this regard, the development of effective approaches to the regeneration of bone defects represents one of the key goals in modern medicine [2]. New strategies for bone tissue regeneration focus on the use of biodegradable/bioresorbable and biocompatible porous matrices (scaffolds), sometimes with pre-seeded stem cells [3]. To date, several types of scaffolds have been developed to retain cells iin situ, recreate their biological microenvironment, and maintain growth prior to integration into the transplant area [4,5,6]. In particular, porous bioceramics [7], matrices based on natural [8,9,10,11] and synthetic polymers [12,13], and composites [14,15] are under study as scaffolds for bone tissue repair.

In addition to biocompatibility, the in vivo performance of the scaffolds depends on several key factors such as porosity, mechanical and osteoconductive or osteoinductive properties [16,17]. It is known that porosity must be not less than 60% but in an ideal case around 90% [18] to provide the accessibility to cell migration inside the matrix and further tissue ingrowth and formation of the vessels. At the same time, the mechanical properties of the scaffold must be suitable to serve as temporary support until the growing tissue is able to independently withstand the mechanical stress typical to bone tissue. In turn, osteoconductive or osteoinductive properties stimulate cell adhesion, proliferation, differentiation, biomineralization and formation of the bone extracellular matrix.

Biodegradable synthetic polymers from the group of aliphatic polyesters, such as poly(lactic acid) (PLA), poly(lactic acid-*co*-glycolic acid) (PLGA) and poly(ε-caprolactone) (PCL), as well as their composites with inorganic fillers (e.g., hydroxyapatite, cerium oxide, etc.) [19,20], nanocrystalline cellulose [10,15,21], graphene derivatives [22,23], etc. are widely investigated as scaffolds for bone regeneration. There are two main approaches to producing porous scaffolds from aliphatic polyesters. These are thermally induced phase separation (TIPS) [24] and additive manufacturing [25]. In the first case, the material is formed by cooling the metastable polymer solution to a low temperature in the region of instability to induce the de-mixing of a homogeneous polymer solution to separated polymer and solvent(s) phases. The main advantage of this technique is the possibility to produce a highly-interconnected supermacroporous polymer network by a simple and quite fast process. Such materials usually have pores larger than 100 µm, the walls of which are pierced with smaller pores of about 10–20 µm and 0.5–3 µm [26]. This method can be successfully applied to obtain polymer composite materials, but it is limited to the efficient preparation of scaffolds loaded with small molecules. The bottleneck of the TIPS technique is the selection of an appropriate solvent mixture to prepare the initial metastable polymer solution providing the final distribution of small molecules in the polymer matrix but not in a solvent phase.

Additive manufacturing, or 3D printing, is based on the layer-by-layer addition of material to create a modeled 3D-structure [27,28]. This method provides the ability to produce matrices of the selected design and composition from thermoplastic polymers and nanofillers or small molecules stable at printing temperature. Moreover, it is a simple and solvent-free technique. The comparison of the mechanical properties of polymer scaffolds prepared by TIPS and 3D printing techniques revealed the superiority of the latter [26]. This fact can be explained by the less homogeneous structure and higher total porosity of the TIPS materials, which, in turn, increase the fragility of such matrices compared to 3D-printed ones.

One of the existing postoperative obstacles is infections and inflammatory effects caused by implantation [29]. The standard preventive treatment for postoperative infections and inflammatory effects is the systemic administration of antibiotics or anti-inflammatory drugs. However, sometimes formed bacterial biofilms or inflamed tissues around the implant do not provide the necessary permeability for systemically administered drugs, which in turn contributes to a decrease in therapeutic efficacy. An alternative pathway to prevent such implant-related drawbacks is to impart to the implanting scaffold antimicrobial and/or anti-inflammatory properties. This can be achieved by localizing the appropriate drugs on the surface of the scaffold [30,31] or by their introducing into the material matrix to provide local treatment [32,33].

Currently, there are a number of papers reporting the preparation of materials with antimicrobial properties for different biomedical tasks. For instance, among them ciprofloxacin-modified polyurethane/PLA porous scaffolds for regeneration of skin [33], amoxicillin-loaded laponite-doped PLGA nanofibers [34], ciprofloxacin-loaded laponite-doped PCL nanofibers [35], ciprofloxacin-loaded PCL/poloxamine hot melt blends [36], ciprofloxacin-loaded PCL/hydroxyapatite composite films [37] and dexamethasone-loaded porous PLGA and PCL scaffolds produced by compressed CO_2_ foaming and proposed for bone regeneration [38]. As to 3D-printed materials, Radhakrishnan et al. recently reported the fabrication of PCL scaffolds with antimicrobial properties provided by incorporated silver nanoparticles [39]. In turn, Zhou et al. developed a 3D-printed PCL scaffold with vancomycin-loaded PLGA microspheres adsorbed on their surface. In this case, the authors initially prepared vancomycin-loaded PLGA microspheres by double emulsion method and then adsorbed them onto a 3D-printed polydopamine-coated PCL matrix [40].

Dexamethasone-containing scaffolds as materials with anti-inflammatory properties have been prepared by Lee et al. [41] and Sun et al. [42]. The first group prepared the 3D PCL scaffold with a combination of electrospinning and 3D printing techniques for application in tracheal replacement [41]. The dexamethasone was adsorbed on the surface of the modified scaffold. Another group developed the scaffolds by selective laser sintering of dexamethasone-loaded PLA microspheres [42].

Here, we report the development, characterization and in vitro biological evaluation of PCL scaffolds loaded with ciprofloxacin (CIP) or dexamethasone (DEX). In contrast to known approaches to producing scaffolds with antibacterial or anti-inflammatory properties, the novelty of this study is based on the direct introduction of a drug into a 3D printing process using a polymer-drug blend. PCL was selected as the matrix polymer due to its lower melting point in comparison with PLA (60–70 °C vs. 160–180 °C) [43,44]. The lower printing temperature is favorable to preserve drugs from temperature-driven degradation. Moreover, PCL causes a less inflammatory reaction in vivo compared to PLA because of slower degradation and less acidification of the implant area [45].

A series of scaffolds containing CIP or DEX was 3D-printed and examined for mechanical properties in a compression test. The effect of the additives on the mesoporosity and specific surface area of the scaffolds and, in turn, the rate of further drug release, was established and analyzed. CIP and DEX release profiles were approximated with a number of mathematical models to evaluate the mechanism of drug release. In vitro biological evaluation was performed to explore the cytotoxicity, antibacterial and anti-inflammatory properties of the 3D-printed PCL/CIP and PCL/DEX scaffolds.

## 2. Materials and Methods

### 2.1. Chemicals, Supplements and Biologicals

Tin (II) octoate (SnOct_2_) (92.5–100%), *ε*-caprolactone (97%), ciprofloxacin hydrochloride (CIP, ≥98%), dexamethasone (DEX, ≥98%), lipase from *Candida rugosa* (1300 U/mg), and salts of analytical grade of purity for buffer solution preparation were purchased from Sigma–Aldrich (Darmstadt, Germany). Organic solvents such as chloroform, methanol (MeOH) and tetrahydrofuran (THF) were supplied by Vecton Ltd. (St. Petersburg, Russia) and distilled prior to use according to standard protocols for their purification.

Poly(*ε*-caprolactone) (PCL) was synthesized in bulk by ring-opening polymerization of *ε*-caprolactone as described previously [46] using MeOH as a co-initiator. The ratio of [MeOH]:[SnOct_2_]:[monomer] was 2:1:5000. Weight average molecular weight (*M_w_*) and dispersion (*Đ*) of PCL were determined by size-exclusion chromatography (SEC) in THF at a flow rate of 1.0 mL/min and 40 °C. SEC analysis was carried out using a Shimadzu HPLC system (Shimadzu, Tokyo, Japan) consisting of a pump LC-10AD VP, a system controller SCL-10A VP, a refractometric detector RID-10A, a CTO-20A column thermostat and a Rheodyne 725i injection valve (Rohnert Park, CA, USA) and equipped with two columns of Agilent PLgel MIXED-D (7.5 mm × 300 mm, 5 µm, Agilent, Santa-Clara, CA, USA). Polystyrene standards (2000–450,000, Waters, Milford, MA, USA and Agilent Technologies, Santa Clara, CA, USA) were used for column calibration. Data processing was carried out using LC Solution Shimadzu software (vs. 1.25, Shimadzu, Kyoto, Japan). The intrinsic viscosity (*η*) of PCL was determined using an Ostwald’s capillary viscometer in CHCl_3_.

Hydrophobic PTFE syringe filters with a pore size of 0.22 µm and a diameter of 13 mm were purchased from Nantong FilterBio Membrane (Nantong, Jiangsu, China).

Human fetal mesenchymal stem (FetMSC) and human osteosarcoma (MG-63) cell lines used for cytotoxicity assessment (MTT test) were received from the Vertebrate Cell Culture Collection of Institute of Cytology RAS (St. Petersburg, Russia). FetMSC and MG-63 cells were cultivated as described in our recent publications, respectively [46,47]. Human monocytic leukemia cells (THP-1 cells) were used to study in vitro effects of PCL/DEX scaffolds. THP-1 cells were received from the Cell Culture Collection of the Institute of Experimental Medicine (St. Petersburg, Russia). The THP-1 cells were cultivated at 37 °C in a humidified 5% CO_2_ atmosphere in RPMI 1640 medium (Biolot, St. Petersburg, Russia) containing 10% (*v*/*v*) thermal inactivated fetal bovine serum (FBS, Gibco, Life Technologies, Paisley, UK), 2 mM *L*-glutamine (Biolot, St. Petersburg, Russia) and 50 μg/mL gentamicin (moisture < 5%, Biolot, St. Petersburg, Russia) as it was described previously [48].

The antibacterial effect was studied with the use of *Pseudomonas aeruginosa* (*P. aeruginosa*), bacterial strain ATCC 27853, received from the Museum of microbiological cultures of Institute of the Highly-Pure Biopreparations (St. Petersburg, Russia). Before the experiments, the bacterial culture was grown in the Mueller-Hinton broth (MHB, HiMedia, Thane, Maharashtra, India).

### 2.2. Manufacturing of 3D-Printed Scaffolds Loaded with CIP or DEX

In order to manufacture scaffolds containing drugs distributed directly in the PCL matrix, CIP or DEX in amounts of 0.5, 1 or 5 wt% were blended with a polymer melt at 80 °C in an air thermostat using a Teflon cup and a glass rod. After that, the cooled composite blend was additionally blended using the micro scientific bench top two roll mills (LRM-M-100, Labtech Engineering Co. Ltd., Samutprakarn, Thailand) using temperatures of 40 and 55–60 °C on one and the other roll, respectively, with a speed drive of about 10 rpm of each roll. After cooling, the blends were loaded into a 3D printer (BioScaffolder 3.2, GeSim, Radeberg, Germany) equipped with a heating stage and a pneumatic extruder. Hexagonal porous scaffolds 6 mm in diameter and 1.1 mm (three-layered) or 1.8 mm (five-layered) in height were printed by layer-by-layer deposition using a 0.4 mm tip. GeSim Robotics software (vs. 1.16.0.3892, GeSim, Radeberg, Germany) was used to create the 3D printing model. To obtain uniform scaffolds, the following optimal printing settings were applied: cartridge temperature 80 °C, pneumatic extruder pressure 500 kPa, printing head speed 0.8 mm/s, height of one layer 0.37 mm, distance from the printing head to the printed layer 0.60 mm, pause at layer printing start 0.8 s, horizontal print head “take-off” motion, glass substrate temperature 35 °C. The following series of scaffolds were manufactured: PCL as control, PCL/DEX containing 0.5, 1 or 5 wt% dexamethasone (PCL/DEX-0.5, PCL/DEX-1 and PCL/DEX-5) and PCL/CIP containing 1 or 5 wt% ciprofloxacin (PCL/CIP-1 and PCL/CIP-5).

The images of the surface of pristine and composite polymer 3D matrices were taken with a Nikon Eclipse E200 (Tokyo, Japan) optical microscope equipped with a U3CMOS digital camera using the Nikon software NIS-Elements (Tokyo, Japan).

To confirm the composition of the 3D-printed matrices, Fourier-transform infrared (FTIR) spectroscopy was performed with the use of an IRAffinity-1S spectrometer (Shimadzu, Tokyo, Japan). The FTIR spectra of individual components (PCL, drugs) and composite materials distributed in a 10 mg KBr pellet containing 2 wt% of the composite and 1 wt% of the drug (CIP or DEX) were recorded in the range of 500–4000 cm^–1^. The spectral resolution and the number of scans per sample were 2 cm^−1^ and 40, respectively.

### 2.3. Compression Test

The mechanical properties of the 3D-printed five-layer matrices were examined in the compression tests. The investigation was performed at room temperature in the uniaxial compression mode using the AG-100X Plus universal unit (Shimadzu Corp., Kyoto, Japan) at a compression rate of 1 mm/min. The test was stopped when the degree of compression of the sample reached 70%. The following characteristics of the materials were determined: compression modulus (*E*), the yield stress *(σ_y_)*, the maximum compressive strength (*σ_max_*) and the force applied at the maximum compression (*F_max_*). These parameters were calculated without taking into account the porosity of the material, by dividing the corresponding forces by the total area of the specimen prepared using the 3D printing method.

The compressive strength and compression modulus were also measured for monolithic specimens prepared by hot molding at 105 °C in the form of cylinders with a diameter of 10 mm and a height of 3 mm. Compression was carried out at a speed of 1 mm/min up to the maximum compressive deformation of 80%. Examples of deformation curves for 3D-printed scaffolds based on PCL and its composites containing 5 wt% of the drug are shown in Figure S1 (Supplementary Materials).

### 2.4. Measurements of Specific Surface Area and Porous Characteristics

The specific surface area and mesopore volume of the 3D-printed specimens were assessed by a nitrogen gas sorption analyzer (NOVA 1200, Quantachrome, Germany), and were determined by a Multipoint BET (Brunauer–Emmett–Teller) method. The sample was degassed before the measurements by nitrogen flow under reduced pressure. The DFT (Density Functional Theory) method was applied to calculate the pore characteristics from the analysis of the desorption branches of the isotherms. Some histograms of pore distribution are shown in Figure S2 (Supplementary Materials).

### 2.5. Drug Release

The rate of drug release from the scaffolds was studied as follows. A specimen of 3D-printed three-layer scaffold containing 1 or 5 wt% of CIP or 0.5, 1 or 5 wt% of DEX was immersed in 1 mL of 0.1 M phosphate buffer solution (PB, pH 7.4) or in PB supplemented with *Candida rugosa* lipase enzyme (3 mg/mL). All specimens were incubated at 37 °C under orbital stirring (150 rpm). After certain time intervals, the solution was completely removed and replaced with a fresh one. The collected solutions were filtered through syringe PTFE filters and analyzed spectrophotometrically (SF-56 SPECTR LLC, St. Petersburg, Russia). The absorbance was measured at 272 nm for CIP and 242 nm for DEX analysis. In spectrophotometric analysis, 0.1 M PB (pH 7.4) was used as the control solution when the release was studied in buffer solution, and the same buffer with lipase when the release was studied in enzyme-containing buffer solution. The drug quantity was determined regarding the calibration plot preliminary built for each drug. The release experiments were carried out in three independent series for 45 days.

To determine the content and reproducibility of drug loading from scaffold to scaffold, the complete degradation of scaffolds containing drugs was performed by adding 1 mL of 1 M KOH solution to the specimen and incubating for 12 h in an orbital shaker at 37 °C and 150 rpm with periodic heating in a water bath at 70 °C. The contents of CIP or DEX in the solutions, filtered through a PTFE syringe filter after complete scaffold degradation, were determined spectrophotometrically as described above. The release experiments were carried out in three independent series for each scaffold composition.

A number of mathematical models (Table 1) were applied for CIP and DEX release profile approximation in order to analyze the driving forces of drug release [49]. A 31-h drug release process was taken for modeling because it allowed us to stay within the 60% cumulative release values. DDSolver add-in for Microsoft Excel developed by Yong et al. was used for plotting the regression lines and evaluation of model parameters [50]. The obtained correlation coefficients were taken as a value indicating that the model fits the release profile. Kinetic constants and model parameters gave the information on release rates and limiting factors of release.

### 2.6. Cytotoxicity

FetMSCs were cultured in a CO_2_ incubator at 37 °C in a humidified air atmosphere containing 5% CO_2_ in DMEM/F12 medium (Dulbecco’s modified Eagle’s medium) containing 1% essential amino acids, 10% (*v*/*v*) thermal inactivated FBS (HyClone, Logan, UT, USA), 1% L-glutamine, 50 U/mL penicillin, and 50 μg/mL streptomycin (Sigma–Aldrich, Darmstadt, Germany).

Dry three-layer scaffolds were sterilized under UV exposure for 15 min prior to the examination. For the experiment, the scaffold specimens were placed into wells. Then, cells in 100 µL of culture medium were added into each well containing a specimen. 5 × 10^3^ cells/well or 3 × 10^3^ cells/well were seeded into 96-well plates containing test specimens and cultured for 24 or 120 h, respectively. The plastic bottom of the wells in the culture plate was used as a control surface.

At the end of the incubation period, the medium was removed and 50 μL/well of DMEM/F12 medium with MTT reagent (Sigma, St. Louis, MO, USA, 0.1 mg/mL) was added. The cells were incubated in a CO_2_ incubator for 2 h at 37 °C. After removal of the supernatant, formazan crystals formed by metabolically viable cells were dissolved in dimethyl sulfoxide (50 μL/well) and transferred to the clean wells for measurement of optical density at 570 nm using a flatbed spectrophotometer (ThermoFisher Multiscan Labsystems, Waltham, MA, USA). All cell experiments were carried out for a series of 3 scaffolds of the same composition. The results are given as mean value ± SD.

### 2.7. Osteodifferentiation

FetMSCs were cultured in a CO_2_ incubator at 37 °C in a humidified atmosphere containing air and 5% CO_2_ in DMEM/F12 culture medium (containing 1% essential amino acids, 10% (*v*/*v*) thermal inactivated FBS (HyClone, Logan, UT, USA), 1% L-glutamine, 50 U/mL penicillin, and 50 μg/mL streptomycin (Sigma–Aldrich, Darmstadt, Germany). After achieving a monolayer (2–3 days until confluence 90%), the medium was changed to MSCg 05–440-1B one (Biological Industries, Beit–Haemek, Israel) containing 10% (*v*/*v*) thermal inactivated FBS (HyClone, Logan, UT, USA), 1% L-glutamine, 50 U/mL penicillin and 50 μg/mL streptomycin, β-glycerophosphate, dexamethasone (1000×), and ascorbic acid (Sigma–Aldrich, Darmstadt, Germany). For alkaline phosphatase staining, after 14 days of cultivation, cells were washed three times with 1xPBS and fixed with 4% formaldehyde solution for 30 min at room temperature. The cells were washed with PBS three times for 5 min each, then were treated with BCIP-NBT (5-bromo-4-chloro-indolyl phosphate tetrazole blue, Sigma, Darmstadt, Germany) solution for 30–60 min in the dark at room temperature.

In addition, staining with alizarin red S was performed to evaluate the biomineralization of the scaffolds. For this purpose, cells were cultured the same way as in the experiment with alkaline phosphatase, but for 28 days. At the end of cultivation, the medium was removed, specimens were washed twice with PBS, and fixed with 70 % ethanol for 30 min and then washed twice with deionized water. After water removal, a solution of Alizarin Red S (Sigma–Aldrich, Darmstadt, Germany, 40 mM in deionized water) was added and incubated for 20 min.

All specimens were washed three to four times with deionized water, air dried and analyzed in the transmitted light with the use of Eclipse E200 optical microscope with U3CMOS digital camera (×4) and NIS-Elements software (Nikon, Tokyo, Japan).

### 2.8. Antibacterial Properties of PCL/CIP

A culture of *P. aeruginosa* stored at −70 °C was used to prepare a daily bacterial suspension. A frozen suspension (1.0 mL) with a titer of ~10^9^ CFU/mL was added to 50 mL of MHB and cultured at 37 °C under shaking (160 rpm) for 18 h until the logarithmic growth phase. The suspensions of *P. aeruginosa* with a titer of 10^7^ and 10^6^ CFU/mL were prepared from a suspension of daily *P. aeruginosa* with a titer of 8.2 × 10^9^ CFU/mL. To evaluate the inhibition of bacterial growth of *P. aeruginosa*, 100 µL of the obtained suspensions were plated on Luria–Bertani (LB) agar in Petri dishes (*d* = 10 cm), and after drying the test three-layer scaffolds (PCL/CIP-5 and PCL as control) were placed. The Petri dishes were covered with cups and incubated at 37 °C for 24 h. After the given time, the inhibition zones were measured. The diameter of inhibition zones was calculated as mean value ± SD (*n* = 4).

To evaluate the bactericidal activity of the scaffold specimens, *P. aeruginosa* suspensions with titers of 10^7^ and 10^6^ CFU/mL were poured into the tubes and the PCL scaffolds containing CIP and not-containing it were put into the tubes (*n* = 3 for each scaffold type). After 24 or 48 h, the suspensions were titrated in sterile saline solution and the corresponding dilutions were plated on LB agar for 24 h to determine colony-forming units (CFU).

### 2.9. Anti-Inflammatory Activity of PCL/DEX

Primarily, the effects of PCL/DEX scaffolds on cell viability were investigated. THP-1 cells were incubated in the presence of different types of PCL scaffolds in 96-well flat-bottom culture plates (Sarstedt, Germany) for 24 h. Next, THP-1 cells were gently resuspended and without extra manipulations were transferred to 75 mm × 12 mm flow cytometry tubes (Sarstedt, Germany) and stained for cell viability. For this purpose, YO-PRO-1 iodide (final concentration 250 nM; Molecular Probes, Eugene, OR, USA) and propidium iodide (PI, final concentration 1 μM, Merck KGaA, Darmstadt, Germany) staining were utilized. Unfilled PCL scaffolds were used as a control. Method principals and “gaiting strategy” were described previously [51]. At least 10^4^ single THP-1 cells were analyzed per each sample. Flow cytometry data were obtained with a Navios™ flow cytometer (Beckman Coulter, Beckman Coulter Inc., Indianapolis, IN, USA) equipped with 405, 488, and 638 nm lasers and analyzed using Navios Software v.1.2 and Kaluza™ software v.2.0 (Beckman Coulter, Beckman Coulter Inc., Indianapolis, IN, USA). The data were presented as the percentage of viable cells per sample ± SD.

Next, the ability of PCL/DEX scaffolds to down-regulate THP-1 (human monocytes) cell activation was investigated. THP-1 cells were activated in vitro by adding the recombinant human tumor necrosis factor-α protein (TNFα, final concentration 2 ng/mL, BioLegend Inc., San Diego, CA, USA), while untreated THP-1 cells were used as a negative control. The PCL/DEX-1 scaffolds, as well as, negative (unfiled PCL scaffolds) and positive (DEX solution in final concentrations 5–100 μg/mL) controls were added to 200 μL of THP-1 cell suspension (200 µL of cell culture medium containing 1 × 10^5^ cells in suspension) and incubated in 96-well flat-bottom culture plates (Sarstedt, Nümbrecht, Germany) for 24 h. Then the cells were transferred to 75 mm × 12 mm flow cytometry tubes (Sarstedt, Nümbrecht, Germany) and washed with 4 mL sterile PBS (centrifugation at 300× *g* for 5 min). The obtained cell sediments were resuspended in 100 µL of fresh PBS and stained with mouse anti-human CD54-PE antibodies (Beckman Coulter Inc., Indianapolis, IN, USA) for 15 min in the dark as described previously [52]. Finally, THP-1 cell samples were washed one more time and stained with DAPI solution (final concentration 1 μg/mL, BioLegend Inc., San Diego, CA, USA) to differentiate between live and dead cells for viability. At least 10^4^ single THP-1 cells per sample were acquired. Flow cytometry data were obtained with a Navios™ flow cytometer. The intensity of CD54 expression was ultimately measured as mean fluorescence intensity (MFI) on the cell surface of viable THP-1 cells.

### 2.10. Statistics

The mechanical tests, cytotoxicity and osteodifferentiation studies of each composition specimens were tested three times. Antibacterial properties were evaluated in a series of four specimens of each composition. In all cases, the data are presented as mean value ± SD. Student’s *t*-test was used to analyze the statistical significance of the results.

Anti-inflammatory properties were performed in three independent series with the use of at least three specimens of each composition in a series. In all cases, the data are presented as mean value ± SD. In this case, the non-parametrical Mann–Whitney U test was used to analyze the statistical significance of the results.

In all cases, the results are considered statistically significant if *p* < 0.05.

## 3. Results and Discussion

### 3.1. Manufacturing and Characterization of Drug-Loaded 3D-Printed Scaffolds

**Figure 1:**
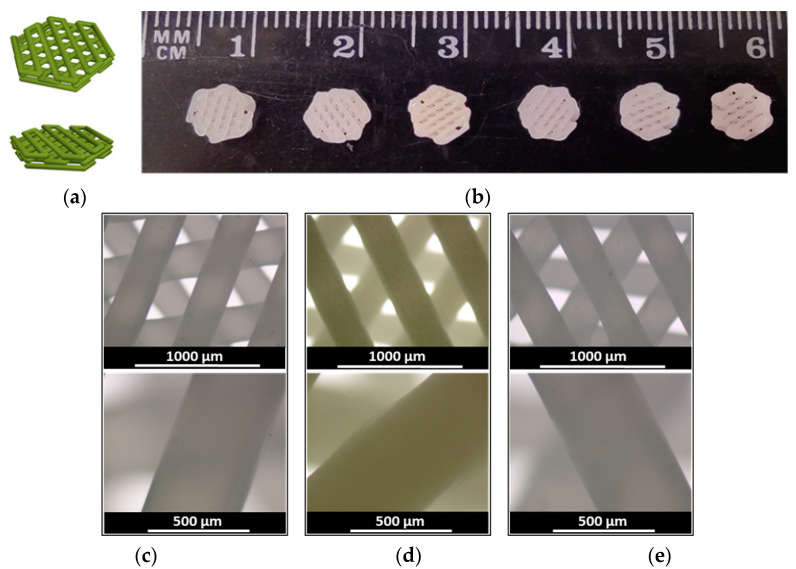
3D-printed three-layer scaffolds: (**a**) 3D model; (**b**) 3D-printed scaffolds based on (from left to right) PCL, PCL/CIP-1, PCL/CIP-5, PCL/DEX-0.5, PCL/DEX-1, and PCL/DEX-5, respectively; optical microscopy images: (**c**) PCL; (**d**) PCL/CIP-5; and (**e**) PCL/DEX-5 at ×4 (top row) and ×10 (bottom row) magnification.

PCL (*M_w_* = 103,000, *Đ* = 1.7; *η*_(CHCl3)_ = 1.40 dL/g) was used for the manufacturing of scaffolds loaded with antibiotic (CIP) or anti-inflammatory drug (DEX) via layer-by-layer fused deposition using a 3D printer. Before 3D printing, CIP and DEX were blended in the PCL melt at 80 °C. The 3D-printing was carried out at the temperature when PCL was melted (80 °C). CIP and DEX have melting points at 326 [36] and 262–264 °C [53], respectively, and are thermally stable at printing temperature. The 3D-printing was fulfilled using a setup protocol previously optimized for printing composite scaffolds of PCL with nanocrystalline cellulose (NCC) [54] (Section 2.2). The only difference was that in this work, the height of one layer was 0.37 mm, but not 0.33 mm as previously used for PCL/NCC composites. The average mass of a 3D-printed scaffold was 18.0 ± 0.3 mg with a total porosity of 58 ± 1%. The drug loading was 1 and 5 wt% for PCL/CIP (PCL/CIP-1 and PCL/CIP-5), and 0.5, 1 and 5 wt% for PCL/DEX (PCL/DEX-0.5, PCL/DEX-1 and PCL/DEX-5). The images of the 3D-printed scaffolds are shown in Figure 1.

The dosage of the drugs in the implantable material is essential for successful therapy. In this regard, it is important to determine the content and reproducibility of the loaded drugs in the polymer mixture prepared by 3D printing. Based on the results of complete degradation conducted, the amounts of drugs determined spectrophotometrically in hydrolysates were as follows: 85 ± 2% and 84 ± 6% for PCL/DEX-1 and PCL/DEX-5, respectively; 89 ± 8% and 91 ± 4% for PCL/CIP-1 and PCL/CIP-5, respectively.

FTIR spectroscopic analysis revealed the presence of a number of characteristic bands corresponding to groups and bonds of individual substances in the FTIR spectra of 3D-printed composite matrices (Figure S3, Supplementary Materials). For the PCL/CIP composite, the bands at 3087, 3044 and 3014 cm^−1^ correspond to symmetric and asymmetric stretching vibrations C-H in =CH and in the cyclopropyl group (CH_2_) of CIP [55]. The band at 1617 cm^−1^ refers to asymmetric vibrations of ketone (C=O), which is shifted to the far infrared region due to the deprotonation of the carboxyl group accompanied by the formation of a zwitterion [35,55,56]. Finally, the bands at 1589 and 1498 cm^−1^ correspond to the C=C stretching vibrations of quinolone moiety [57,58]. For PCL/DEX, the characteristic bands at 1665 and 1622 cm^−1^ refer to the carbonyl (C=O) stretching vibrations [59,60,61] and the double bond (C=C) stretching vibrations [62,63], respectively. FTIR spectra of both types of composites also show some drug-specific frequencies in the fingerprint region (1500–500 cm^−1^). The absence of significant changes in the drug spectra in the composites (broadening of peaks, disappearance of any significant signals, etc.) relative to the spectra of individual drugs indicates that DEX and CIP were mechanically integrated with the PCL matrix without specific drug-matrix interactions or drug destruction during 3D printing.

It is known that bone tissue must withstand strong compression, therefore testing the mechanical properties of materials for bone tissue regeneration is very important [64,65]. Given that the mechanical properties of non-porous materials and porous scaffolds can differ, both types of matrices were examined.

**Figure 2:**
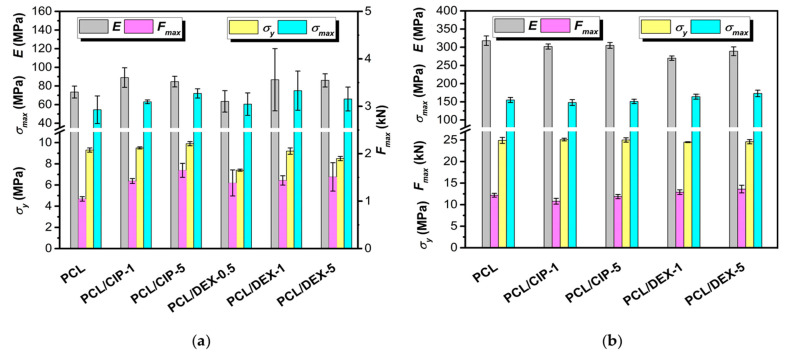
Compression properties of unfiled PCL and PCL/drug matrices: (**a**) 3D-printed five-layer porous scaffolds; (**b**) cylindrical non-porous materials. Abbreviations: *E* is the compression modulus, *σ_y_* is the yield stress, *σ_max_* is the maximum compressive strength, and *F_max_* is the force applied at the maximum compression.

Compression tests for 3D-printed matrices were performed until the compression ratio of the sample reached 70%. All specimens withstood successfully the compression up to the maximal deformation value. The determined compression modulus (*E*) for the pure 3D-printed PCL scaffolds was 74 ± 6 MPa. This value is close to the result obtained by Ma et al. for the 3D-printed PCL scaffolds. The determined compression modulus was reported to be 72 ± 11 MPa [66]. The same authors showed that the introduction of a polymer (poly(vinyl acetate), PVAc) or hydroxyapatite (HA) into the PCL matrix by 3D printing affected the compression modulus [66]. It increased when HA was used and decreased when PVAc was a filler. In our case, the introduction of both drugs, which are small molecules, had no noticeable effect on the mechanical properties of PCL (Figure 2a).

Furthermore, the compression tests were performed for monolithic cylindrical specimens obtained by hot molding of PCL and its composites with CIP or DEX. The experiments were carried out under a maximum compressive deformation of 80%. As for scaffolds, it was found that the introduction of both drugs into PCL with varying content did not affect their mechanical characteristics (Figure 2b). Thus, no statistical difference in the mechanical properties of both scaffolds and monolithic hot-molded cylinders was observed when 0.5, 1 or 5 wt% of the drugs were added to the PCL matrix.

Earlier, Liu et al. established the reduction in the compression modulus of porous HA-filled PCL-based 3D materials compared to non-porous composites [14]. Specifically, the authors observed a decrease in *E* from 341 MPa for non-porous monolithic material to 46 MPa for a porous 3D-printed scaffold. Recently, Averianov et al. revealed a decrease in *E* from 350–380 MPa for PCL-based monolithic composites filled with modified nanocrystalline cellulose to 120 MPa for 3D-printed scaffolds of the same composition. Thus, lower mechanical properties observed in this work for 3D-printed scaffolds (E ~ 70–90 MPa) compared to non-porous hot-molded monoliths (~300 MPa) were expected due to the presence of voids in the 3D-printed matrices. Nevertheless, the values of compression modulus found in the present study are suitable for repairing defects in some bones, for example, trabecular one, whose compression modulus is close to 12 MPa [67].

In addition, the effect of drugs as additives on the porous characteristics and specific surface area was analyzed. For this purpose, the pore volume, average pore size and specific surface area were determined by BET analysis (Table 2). The filament walls in all scaffolds were pierced with mesopores. The average pore size in PCL or PCL scaffolds containing a low amount of drug (1 wt% CIP or 0.5 wt% DEX) was about 3.2–3.3 nm, and the pore size distribution was in the range of 2.8–20 nm. In turn, an increase in the drug content was accompanied by an increase in the average pore size to 4.5 nm with a simultaneous decrease in the pore size distribution to 3–11 nm. At the same time, when DEX was introduced into the PCL matrix and its loading was increased from 0.5 to 5 wt%, a decrease in pore volume and specific surface area was detected.

In turn, the low addition of CIP (1 wt%) contributed to an increase in porosity and specific surface area, while an increase in CIP loading had the same effect as DEX. Similar trends were also observed by other research groups. In particular, Costa et al. observed a decrease in the porosity of 3D-printed scaffolds when poloxamine was introduced into the PCL matrix and the filler content was increased [68]. Recently, Iga et al. reported the preparation of polyurethane/PLA scaffolds containing CIP by TIPS [33]. A slight increase in porosity was detected by authors at some compositions containing low loads of CIP, while a general decrease in porosity was observed at greater CIP loads.

### 3.2. Drug Release Study

The release of drugs from the matrices was studied in a model buffer solution (0.1 M sodium phosphate buffer, pH 7.4) and the same buffer containing lipase. The latter is an enzyme belonging to the subclass of esterases (EC.3.1.1) [69]. It is known that bone cells and especially osteoclasts are esterase-expressing [70]. In our case, lipase was selected as a model enzyme because of its known activity against aggregated substrates containing ester bonds formed by fatty acids. All scaffolds were incubated for 45 days at 37 °C.

According to our previous results on the biodegradation of 3D-printed PCL scaffolds under the same model conditions, the degradation rate was quite low. Less than 2% of the polymer degraded to monomer within two months [46]. In this study, the established weight loss of neat PCL scaffold after 45 days was close to 2% in a buffer medium containing lipase. In the case of PCL containing 5 wt% drug, the weight loss was 5.3 ± 0.4% in buffer solution and 7.8 ± 0.5% in lipase-containing buffer medium.

**Figure 3:**
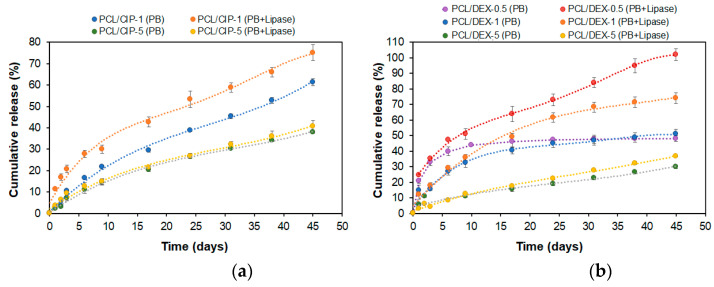
Profiles of cumulative release of CIP or DEX from the scaffolds in 0.1 M sodium phosphate buffer, pH 7.4, or the same buffer containing lipase (37 °C): (**a**) PCL/CIP-1/5; (**b**) PCL/DEX-0.5/1/5. Three-layer 3D-printed scaffolds were used for the release study. The percentage drug content per scaffold corresponded to the following mass drug loading: 0.5 wt% corresponded to 90 μg/scaffold; 1 wt% corresponded to 180 μg/scaffold; and 5 wt% corresponded to 900 μg/scaffold.

The profiles of cumulative release are shown in Figure 3. For scaffolds filled with 1 wt% CIP, the drug release was more intense in the medium containing the enzyme: 75% of loaded CIP was released within 45 days. In turn, CIP release in lipase-free buffer solution did not exceed 61% for the same time. The five-fold increase in the CIP loading resulted in a lower percentage of drug release. In this case, CIP cumulative release was 41% within 45 days. At the same time, the differences in release rate in the enzyme-free and enzyme-containing media were smoothed out.

A similar tendency was also observed for the DEX release. For scaffolds loaded with 0.5 and 1 wt% of DEX, the drug release in the enzyme-containing medium reached 100 and 74%, respectively. At the same time, only 49–51% DEX was released in the enzyme-free buffer solutions for scaffolds with both loadings. As with CIP, increasing DEX loading to 5 wt% resulted in a decrease in the percentage of drug released and a flattening of the effect of the medium on the release rate. The decrease in the DEX release rate with increasing drug loading from 0.5 to 5 wt% may be related to the established decrease in mesoporosity and specific surface area of scaffolds with increasing DEX content (Table 2). A reduction in the specific surface area, in turn, leads to a decrease in the diffusion rate of the drug both in the absence and in the presence of the enzyme in the buffer medium.

According to the literature, a release of 45% CIP from PCL and PCL/laponite composite electrospun fibers was observed after 14 days of incubation of PCL/CIP nanofibers in PBS (pH 7.4) [35]. The addition of laponite to the PCL/CIP nanofibers accelerated the release of the drug. In this case, a 75% release of CIP was detected. Another effect was observed for the release of amoxicillin (AMX) from PLGA and PCL/laponite electrospun nanofibers [34]. For those composite nanofibers, faster release of AMX was observed for PLGA/AMX (100% within 9 days) than for PLGA/laponite/AMX (63% within 14 days), which may be due to the different physicochemical properties of CIP and AMX. The slower release of CIP from PCL observed in our study is explained by a different material design and a thicker matrix from which the drug needs to diffuse as well as by the higher drug loading.

Makinen et al. studied the release of CIP from cylindrical PLGA pellets in a phosphate buffer solution [71]. CIP release from PLGA/CIP pellets containing 8 wt% (PLGA/CIP-8) of CIP reached 39.2 ± 1.7 µg within the first day, and a complete release of CIP was achieved after 110 days. In our case, CIP release from CIP/PCL-5 scaffolds was 33 ± 4 µg which is comparable to the data reported for the PLGA/CIP-8 pellets.

It is known that to cure a bone infection, a sustainable concentration of the drug for 5 weeks is required to suppress infectious post-surgery side effects [37]. According to an in vitvo study of PLGA/CIP-8 pellets performed by Makinen et al., the released CIP accumulated mainly in the bone, while its systemic concentration was significantly lower. Specifically, the authors indicated that after 3 months, the CIP content in bone reached 250 μg/g and then decreased to 2 μg/g over the next 3 months, while the systemic concentration did not exceed 2 ng/mL [71].

The PCL/CIP scaffolds developed in this work exhibit sustained release. The release from the PCL/CIP-1 and PCL/CIP-5 scaffolds after 45 days was 75 and 41%, respectively, which corresponds to 135 and 370 µg CIP or approximately 2.7 and 7.6 µg CIP per day (excluding the first day). The minimal inhibitory concentration (MIC) of CIP against *P. aeruginosa* is reported to be 0.25–0.50 µg/mL [72,73]. Thus, the developed scaffolds can provide the necessary therapeutic concentration for effective local treatment.

As for DEX release, no similar studies were found in the literature to compare the results obtained. A comparison of DEX and CIP release from PCL scaffolds indicates a slightly faster release of CIP in buffer solution, whereas the release rate from enzyme-containing media was comparable. The faster release of CIP may be explained by its better solubility in water compared to DEX.

**Figure 4:**
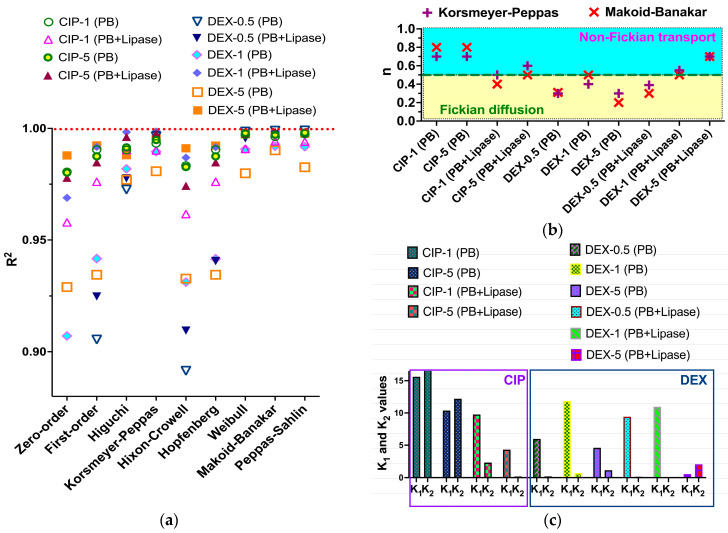
Results of mathematical modeling of CIP and DEX release with application of standard models (Table 1): (**a**) comparison of correlation coefficients of the regressions obtained with different models; (**b**) results obtained by Korsmeyer-Peppas and Makoid-Banakar models application, *n*—model parameter indicating the release mechanism; (**c**) results obtained by application of Peppas-Sahlin model, *K*_1_—impact of diffusional mechanism, *K*_2_—impact of relaxation on release.

A number of mathematical models were applied to approximate the CIP and DEX release profiles in order to assess the mechanism of drug release (Table 1). Different mathematical models are based on various drug release mechanisms, so fitting allowed us to elucidate the factors, which are substantial for CIP and DEX release (Figure 4a and Table S1, Supplementary Materials). Calculated correlation coefficients were used to establish the models, which best fit the release data. Furthermore, an analysis of the rate constants and release exponents parameters allowed us to make some conclusions on factors, which drive drug release (Figure 4b,c).

The analysis of fittings obtained with such basic models as zero-order and first-order showed better correlation coefficients in the latter case. It means that drug release from obtained scaffolds is dependent on the amount of remaining drug in the polymer matrix. A comparison of drug release approximations obtained with the Higuchi and Hixon–Crowell models showed that correlation coefficients are higher in the case of the Higuchi model. Thus, the release process could be considered as controlled by drug diffusion, but not drug dissolution. The fitting of release data with the Hopfenberg model, which is based on polymer erosion, also showed lower correlation coefficients as compared to those obtained with the Higuchi model. Altogether this allows us to conclude that the release of drugs from the scaffolds is more governed by diffusion, rather than drug dissolution of polymer matrix degradation. The only exception is the release of 5% DEX in phosphate buffered solution in the presence of lipase. According to the correlation coefficients found in this case, both drug dissolution and matrix erosion are important factors affecting the release process. Notably, the model, that showed the best correlation with all systems under investigation was the Makoid–Banakar one. This model assumes the total drug release as the result of multiple mechanisms including diffusion, burst release, and controlled release [74].

The Weibull model showed also excellent correlation to the release data. However, this model does not have some physical background and could not be related to some specific mechanism. At the same time, it allows us to elucidate and compare the timescale of the release process (*α*), and to analyze the form of the release curve (*β*). The latter parameter was in all cases below 1, which reveals the parabolic type of the curve, with a high initial slope and consistent exponential character [49,75]. The regression obtained with the Korsmeyer–Peppas model quite well fitted all release data (Figure S4, Supplementary Materials). This allowed to use *n* parameter from this model, which indicates the mechanism of release (Figure 4b). It is known that when the ‘*c*’ value of the Makoid–Banakar model approaches zero, the model becomes identical to the Korsmeyer–Peppas model [76]. As it is observed in our systems (Table S1, Supplementary Materials), the *n* parameter value could be also calculated from the Makoid–Banakar model and used for release mechanism assessment (Figure 4b). The data in the figure show a good correlation of *n* parameter values obtained from different models.

Analysis of the *n* parameter (Figure 4b) revealed that in the case of CIP release from the scaffold in the absence of enzyme, the process is governed by non-Fickian diffusion (*n* = 0.7). The addition of lipase to the release medium changed the release kinetics to Fickian diffusion (*n* = 0.4–0.5). In the case of DEX, we observed the opposite situation, namely, Fickian diffusion (*n* = 0.3–0.5) without enzyme and non-Fickian diffusion (*n* = 0.7) in the presence of the enzyme. The difference might be caused by a difference in the solubility of drugs and their various distribution within the polymer matrices. In the case of more soluble and more hydrophilic CIP, it could be supposed that it is mostly concentrated within the surface layers of the scaffold. Thus, its release in phosphate buffer is affected by swelling of the polymer in such surface layers and possibly in the pores. Despite the hydrophobicity of neat PCL, it could be swelled to a certain extent [43]. Further addition of enzyme could destroy such semi-swelled surface and then only Fickian diffusion of the drug from the inner matrix volume occurs. Another possible situation is that degradation of the PCL on the surface causes the formation of oligomers, which could crystallize and close the nanopores on the surface of materials, thus hindering the transport of drug molecules [77]. This could turn diffusion into Fickian mode. Moreover, the observed effect could be due to the formation of a lipase-based protein layer on the surface of the scaffold.

Oppositely, in the case of DEX, which possesses low solubility in water and greater hydrophobicity the drug is better distributed in the whole volume of the polymer matrix. Thus, the Fickian slow diffusion of the drug within the polymer matrix is the rate-controlling mechanism of release. Degradation of the matrix in phosphate buffer with lipase destroys the surface of the polymer matrix and makes the polymer chains more flexible. This leads to a greater effect of polymer relaxation on hydrophobic drug release. The hydrophobicity of the drug in this case could be the reason for effective drug-polymer interactions, which explain the greater impact of polymer relaxation on DEX release than CIP release after enzyme addition.

The discussed *n* parameter values obtained from Korsmeyer–Peppas and Makoid–Banakar models are in good correlation with the results of the Peppas–Sahlin model application (Figure 4c). This model showed a very nice fitting of experimental release data, so the obtained *K*_1_ and *K*_2_ values, which indicate the impact of diffusion and polymer relaxation, correspondingly, on drug release, could be considered relevant ones. Similarly, to the above-discussed situation, in the case of CIP release in just phosphate buffer, the diffusion (*K*_1_) and polymer relaxation (*K*_2_) have a similar impact on the release rate. After the addition of the lipase, diffusion possesses a greater effect than relaxation. In the case of DEX *K*_1_ is greater than *K*_2_ in all cases except the release of DEX form scaffolds containing 5% of the drug in the lipase-containing medium. In the latter case, the polymer relaxation factor exceeds diffusion one.

Notable that at low DEX content (0.5%) the release is governed mostly by diffusion, both in enzyme-free and enzyme-rich media (Figure 4b,c). Growth of DEX content by an order of magnitude (up to 5%) led to a change in system behavior in medium with lipase, namely, the increased role of polymer relaxation. This revealed the role of drug concentration on drug release peculiarities. It should be also noted that the change in both drugs’ content within the scaffold affects the rate constants of release (Table S1, Supplementary Materials). This observation could be explained by the effect of the drugs on the polymer structuring and porosity, which greatly affect the diffusion of the drugs.

To summarize the modeling part, it could be outlined that the main drug release mechanism from the obtained scaffolds loaded with CIP or DEX is diffusion. However, the drug's hydrophilic/hydrophobic properties and polymer structuring could affect the peculiarities of such diffusion through various effects, such as drug-polymer interaction, polymer swelling, crystallization of oligomeric products of degradation, diffusion in micro- and mesopores, etc. [77].

### 3.3. Cytotoxicity and Osteodifferentiation Study

To validate the biomedical applicability of the developed scaffolds, the cytocompatibility of the 3D-printed materials was evaluated with the use of FetMSC and MG-63 cells. MSCs are usually seeded on the scaffold surface before implantation [54,78]. In this case, the viability of the MSCs during the first 24 h is important to ensure their presence on the scaffold surface for subsequent in vitvo osteodifferentiation. The viability of osteoblasts on the scaffold is important for further induction of collagen-proteoglycan matrix formation by osteoblasts, which, in turn, provides binding of calcium salts and bone tissue formation [79,80]. Unlike quite inert materials containing antibiotics [34], DEX, on the one hand, is known to be toxic to osteoblasts [81,82] and, on the other hand, can induce cell proliferation [42] and osteodifferentiation of MSCs [68].

**Figure 5:**
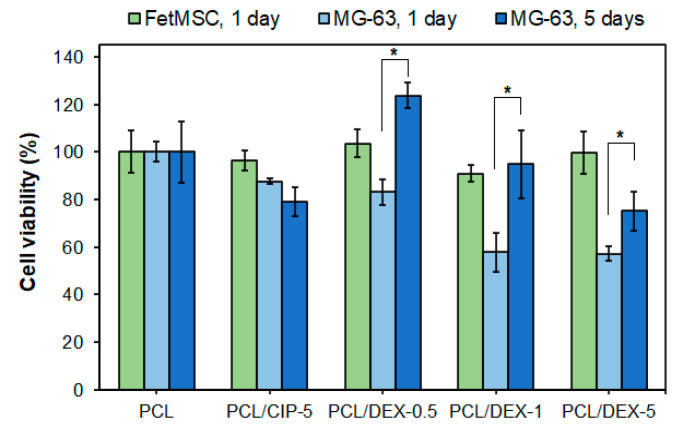
Viability of FetMSC and MG-63 cells adhered on the surface of three-layer PCL, PCL/CIP and PCL/DEX 3D-printed scaffolds (MTT assay). Data are presented as mean ± SD (*n* = 3); * the difference between the groups was significant with *p* < 0.05.

Here, unfilled PCL and PCL/CIP-5, PCL/DEX-0.5, PCL/DEX-1 and PCL/DEX-5 were used as a control and the test materials, respectively. All tested materials demonstrated good biocompatibility with FetMSCs for 24 h (Figure 5). In the case of MG-63 cells, PCL, PCL/CIP-5 and PCL/DEX-0.5 were not cytotoxic, while PCL/DEX-1 and PCL/DEX-5 scaffolds showed obvious cytotoxicity within 24 h of coincubation. The result obtained for PCL/CIP-5 on MG-63 cells for 24 h is comparable to that observed by Puga et al. for PCL/poloxamine/CIP on Saos-2 cells (human osteosarcoma) [36]. However, the reported PCL/poloxamine/CIP scaffolds were slightly toxic to Saos-2 cells after 3 days of co-incubation, whereas our scaffolds showed no cytotoxicity to MG-63 after 5 days. Despite the possible cytotoxicity of some CIP-containing materials in vitro, in vitvo tests have shown that ciprofloxacin does not impair bone formation and fracture healing [71].

The lower cytotoxicity of PCL/DEX-0.5 compared to PCL/DEX-1 and PCL/DEX-1 scaffolds is probably related to the amount of DEX released from the matrix during the experiment. In particular, according to release data obtained (Section 3.2), after 1 day, DEX release from PCL/DEX-0.5 was 18 µg, whereas its release from PCL/DEX-1 and PCL/DEX-5 was 1.5–2 times higher (27 and 36 µg, respectively).

Taking into account that DEX can induce cell proliferation [42], the additional experiment with MG-63 cells was performed within 5 days. It was revealed that after 5 days of coincubation of MG-63 cells with PCL/DEX scaffolds, cell viability was increased for all DEX formulations. Moreover, for PCL/DEX-1 scaffolds, cell viability was equal to the control PCL and PCL/CIP. No statistically significant difference was observed for PCL/DEX-1 and PCL/DEX-5 scaffolds.

**Figure 6:**
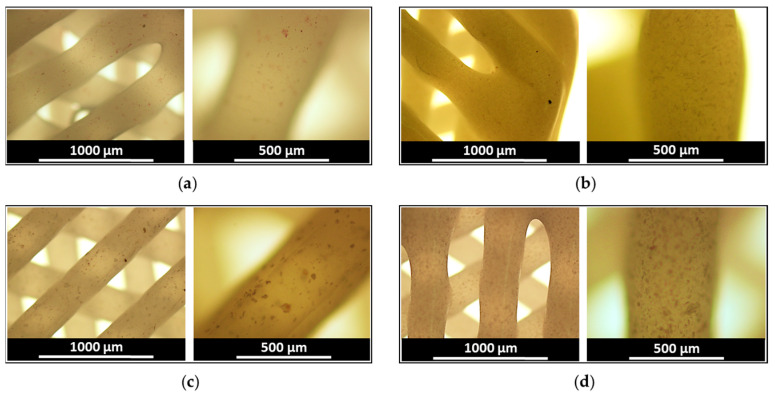
Images of the scaffolds after ALP assay performed on day 15 after the start of osteodifferentiation of FetMSCs in the presence of PCL (**a**); PCL/CIP-5 (**b**); PCL/DEX-1 (**c**); PCL/DEX-5 (**d**) three-layer 3D-printed scaffolds. Magnification ×4 (image on the left) and ×10 (image on the right); slight purple color indicates the presence of ALP.

Alkaline phosphatase (ALP) is one of the markers of the early osteodifferentiation process [83,84]. To evaluate the osteodifferentiation properties of the DEX-containing scaffolds, an ALP assay was carried out after two weeks of incubation of FetMSCs adhered to the PCL/DEX-1 and PCL/DEX-5 matrices. In addition, unfilled PCL and PCL/CIP-5 were also included in the study for comparison. ALP assay revealed a higher number of ALP-positive cells when cells were cultured on scaffolds containing DEX compared to control PCL and PCL/CIP matrices (Figure 6). The obtained result on ALP production was in agreement with the findings previously reported for the composite PLGA/HA/DEX nanofibers [85].

**Figure 7:**
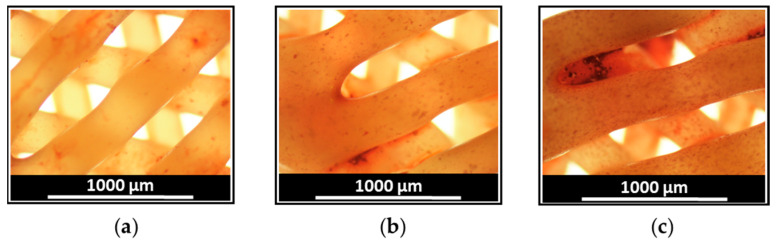
Images of the scaffolds after Alizarin Red S staining performed on day 28 after the start of osteodifferentiation of FetMSCs in the presence of PCL (**a**); PCL/DEX-1 (**b**); PCL/DEX-5 (**c**) three-layer 3D-printed scaffolds. Magnification ×4; red color intensity corresponds to the content of calcium-containing deposits.

Additionally, DEX-stimulated biomineralization was assessed by alizarin red S staining of calcium deposits produced during osteodifferentiation of FetMSCs in the presence of PCL, PCL/DEX-1, and PCL/DEX-5 scaffolds. Since soluble DEX was a component of the medium for osteogenic differentiation, red staining was detected for all scaffolds tested including control (neat PCL). However, the intensity of staining, reflecting the formation of calcium deposits, was higher for DEX-containing scaffolds compared to the control PCL matrix (Figure 7). Moreover, an increase in DEX content from 1 to 5 wt% favored more intense biomineralization of the matrix, as evidenced by more intensive red staining of the scaffolds.

### 3.4. Antibacterial Properties of PCL/CIP Scaffolds

The antibacterial properties of the developed scaffolds were examined against *Pseudomonas aeruginosa*. This bacterial pathogen is one of six more highly virulent and antibiotic resistant bacteria from the ESKAPE (*Enterococcus faecium*, *Staphylococcus aureus*, *Klebsiella pneumoniae*, *Acinetobacter baumannii*, *Pseudomonas aeruginosa*, and *Enterobacter species*) panel [86]. Moreover, hospital-acquired infectious complications caused by *P. aeruginosa* are quite common after the implantation surgery and sometimes are the cause of sepsis syndromes [87,88].

**Figure 8:**
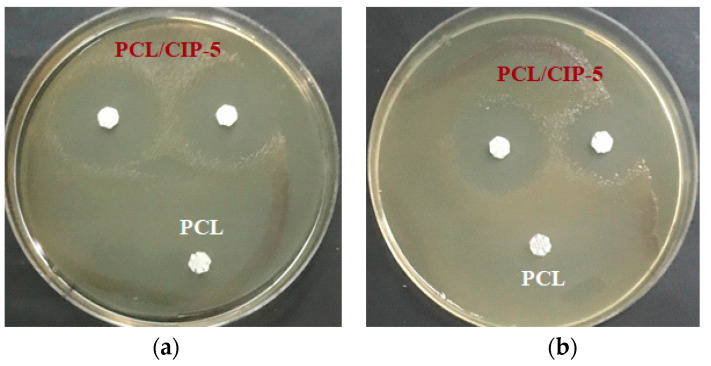
Inhibition of *Pseudomonas aeruginosa* growth by PCL/CIP-5 for 24 h (in agar): (**a**) 10^6^ CFU of *P. aeruginosa*/plate; (**b**) 10^7^ CFU of *P. aeruginosa*/plate. Three-layer 3D-printed scaffolds were used for this assay. PCL was used as a negative control in this experiment as indicated in images (**a**,**b**).

First, the scaffolds were tested using the agar disk diffusion method. For this purpose, PCL/CIP-5 scaffolds and PCL one as a control were placed in agar gels containing 10^6^ and 10^7^ CFU/plate of *P. aeruginosa.* As was mentioned above, MIC of CIP against *P. aeruginosa* (ATCC 27853) is known to be 0.25–0.50 µg/mL [72,73]. CIP release from PCL/CIP-5 scaffolds is 36 µg after 24 h. Thus, the CIP release was sufficient to observe the antimicrobial properties of the scaffolds under study. As expected, PCL did not affect bacterial growth, while scaffolds containing CIP demonstrated a noticeable inhibition of bacteria (Figure 8). After 24 h, the inhibition zones were measured and they were 27.0 ± 0.5 mm and 23.5 ± 0.8 mm (*n* = 4) for 10^6^ and 10^7^ CFU of bacteria per plate, respectively. No change in the diameter of inhibition zones was detected when the time of observation was extended to 48 h.

For instance, Iga et al. reported recently the results of antibacterial testing for the polyurethane/PLA/CIP porous scaffolds containing 2 and 5 wt% of CIP and manufactured by the TIPS technique [33]. The materials were tested in agar against *Staphylococcus aureus*. The diameter of inhibition zones increased with the increasing CIP content and was found to be 16 and 22 mm for 2 and 5 wt% CIP content in the matrix, respectively.

**Figure 9:**
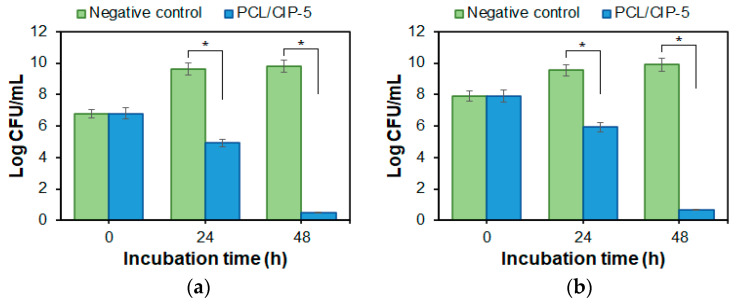
Bactericidal effect of PCL/CIP-5 on *Pseudomonas aeruginosa* in presence for 48 h (in bacterial suspension): (**a**) 10^6^ CFU/mL; (**b**) 10^7^ CFU/mL. Three-layer 3D-printed scaffolds were used for this assay. Data are presented as mean ± SD (*n* = 3); * the difference between the groups was significant with *p* < 0.001.

In addition, the bactericidal properties of PCL/CIP scaffolds were examined via material incubation in *P. aeruginosa* suspensions with a titer of 10^6^ and 10^7^ CFU/mL (Figure 9). A culture without added antibiotics was taken as a negative control. A considerable reduction in bacterial titer was observed within 24 h, while noticeable bacterial growth was revealed in the control series. Specifically, 10^4^ and 10^5^ CFU/mL were counted in the presence of PCL/CIP-5 with an initial titer of 10^6^ and 10^7^ CFU/mL, respectively. At the same time, bacterial growth up to 10^9^ CFU/mL was detected in the control series. Almost complete suppression of *P. aeruginosa* was observed in the case of test specimens at 48 h for both initial titers.

The obtained results are in line with the previously published results. In the study reported by Puga et al. and devoted to the examination of PCL/poloxamine/CIP blends against *S. aureus*, a decrease in titer from 10^6^ to 10^2^ CFU/mL was observed after 24 h. A complete bacterial death was detected after 48 h of coincubation [36].

In general, the antibacterial properties of the developed 3D-printed scaffolds were comparable with previously reported 3D-printed PCL scaffolds filled with AgNPs [39] or PCL/PDA scaffolds bearing adsorbed PLGA microspheres loaded with vancomycin [40]. Despite the comparable antimicrobial properties, PCL/CIP scaffolds lost in mechanical properties in comparison with PCL/AgNPs composites but surpassed the PCL/PDA/PLGA/vancomycin scaffolds in the antibiotic sustained release. Despite the improved mechanics of PCL/AgNPs, the clearance of AgNPs from the body is poorer than that of the released CIP with an unclear release mechanism.

### 3.5. Anti-Inflammatory Properties of PCL/DEX Scaffolds

In order to evaluate the anti-inflammatory properties of DEX-containing matrices, the tumor necrosis factor-alpha (TNFα) was used to model the inflammation effect in THP-1 cells. TNFα is a cytokine produced primarily by monocytes and macrophages, and its primary role is the regulation of immune cells. The systemic overproduction of TNFα activates inflammatory response to infection or tissue damage [89]. It is known also that TNFα stimulates the expression of some TNF receptors (CD20, CD40, CD54, CD120, etc.) on monocytes, lymphocytes, granulocytes and some other cells [90,91,92,93].

Initially, the effect of PCL scaffolds on THP-1 cells was evaluated. Without TNFα treatment, no influence on cell viability was revealed, but a slight decrease in the level of viable cells in the TNFα-activated series was detected (Table 3). The viability of THP-1 cells was reduced in the presence of PCL/DEX-1 scaffold after 24 h in vitro incubation with TNFα-untreated and TNFα-treated cells. An investigation of the effect of DEX on THP-1 cell viability (Table S2, Supplementary Materials) revealed that without TNFα stimulation only high doses of DEX (≥20 μg/mL) contributed to a decrease in cell viability. In turn, TNFα-treated cells even at low doses of DEX (5 μg/mL) noticeably reduced the relative numbers of alive THP-1 cells.

Further, the effects of scaffolds on TNFα-induced expression of CD54 on the surface of THP-1 cells were assessed (Table 4). First of all, the efficacy of TNFα stimulation on CD54 levels on the THP-1 cell surface was confirmed. The expression of CD54 was calculated as the mean fluorescence intensity (MFI). It was equal to 0.77 ± 0.09 in negative control without TNFα treatment and 5.40 ± 1.01 after 24 h in vitro co-cultivation with 2 ng/mL of TNFα (*p* < 0.001). Unfiled PCL and PCL/DEX-1 scaffolds had no effect on CD54 expression by TNFα-untreated THP-1 cells. In turn, both unfilled PCL and PCL/DEX-1 were found to down-regulate significantly the CD54 expression on TNFα-treated THP-1 cells. According to the published data, DEX-containing polymer delivery systems are known to reduce cell-surface expression of CD54 in TNFα-treated cells [52,94]. Indeed, the DEX-containing scaffold demonstrated more pronounced down-regulation of TNFα-induced CD54 expression than the unfiled PCL one (Table 4). A study of the effect of DEX on CD54 expression on the surface of THP-1 cells (Table S3, Supplementary Materials) showed that 25 μg/mL DEX without TNFα stimulation was able to increase CD54 expression after 24 h of co-incubation in vitro. Furthermore, all tested doses of DEX (5–100 μg/mL) effectively reduced CD54 expression on the cell membrane of THP-1 cells.

## 4. Conclusions

In this work, a series of 3D-printed scaffolds based on PCL loaded with CIP or DEX were developed for bone tissue regeneration. The scaffolds were able to withstand compressive mechanical stresses and were characterized by a compression modulus of 80–90 MPa, which is acceptable for the replacement of some bone defects, such as trabecular ones. The release of CIP and DEX in phosphate buffer solution and in the same buffer containing lipase revealed a faster release in the enzyme-containing medium. Drug-loaded PCL scaffolds exhibited sustained release, the rate of which depended on drug content. Higher loading resulted in a lower cumulative drug release percentage which is explained by the decrease in the specific surface area of the PCL scaffolds with increasing drug load in them. The main mechanism of drug release from the obtained CIP- or DEX-loaded scaffolds is diffusion, which in some cases is influenced by various factors related to the polymer matrix. In vitro biological evaluation of the scaffolds containing DEX showed moderate toxicity against osteoblast-like and leukemia monocytic cells. At the same time, PCL/DEX scaffolds demonstrated proliferation and osteodifferentiation properties, which are in agreement with the information known about the properties of DEX. Finally, the preservation of the antibacterial activity of CIP and the anti-inflammatory properties of DEX were confirmed for drug-loaded PCL scaffolds produced by direct additive manufacturing. In particular, it was found that PCL/CIP scaffolds could almost completely kill *Pseudomonas aeruginosa* within 48 h. In turn, PCL/DEX scaffolds demonstrated an effective down-regulation of CD54 expression in experiments on TNFα-activated monocytic cells, respectively.

Overall, the results obtained showed that it is possible to obtain drug-containing scaffolds by direct 3D printing from a polymer/drug blend. It is important that the loaded drug retains its biological activity. However, the pure PCL is quite a hydrophobic polymer, which demonstrates low cell adhesion and rather high inertness in vivo. Moreover, PCL does not possess osteoconductive properties. In this regard, it is reasonable to use PCL-based composite scaffolds containing, in addition to drugs, factors stimulating cell adhesion and biomineralization for future in vivo examination. The combination of drugs and osteoinductive/osteoconductive factors in a single scaffold will allow for enhanced bone repair with minimized post-surgery side effects.

## Figures and Tables

**Table 1:** The list of mathematical models, which were used for CIP and DEX release approximation.

Model	Equation
Zero-order	*F* = *k*_0_ × *t*
First-order	*F* = 100 × [1 − *Exp*(−*k*_1_ × *t*)]
Higuchi	*F* = *k_H_* × *t*^0.5^
Korsmeyer-Peppas	*F* = *k_KP_* × *t^n^*
Hixon-Crowell	*F* = 100 × [1 − (1 − *k_HC_* × *t*)^3^]
Hopfenberg	*F* = 100 × [1 − (1 − *k_HB_* × *t*)*^n^*]
Weibull	*F* = 100 × {1 − *Exp*[−((*t* − *Ti*)*^β^*)/*α*]}
Makoid-Banakar	*F* = *k_MB_* × *t^n^* × *Exp*(−*k* × *t*)
Peppas-Sahlin	*F* = *k*_1_ × *t^m^* + *k*_2_ × *t*^(2 × *m*)^

**Table 2:** Mesoporous characteristics and specific surface area (BET) of 3D-printed scaffolds of different compositions.

Sample	Pore Volume (m^3^/g)	Average Pore Size (nm)	Specific Surface Area (m^2^/g)
PCL	0.002	3.32 ± 0.19	1.78 ± 0.10
PCL/CIP-1	0.005	3.17 ± 0.13	4.98 ± 0.20
PCL/CIP-5	0.001	4.54 ± 0.26	0.59 ± 0.03
PCL/DEX-0.5	0.001	3.16 ± 0.17	0.95 ± 0.05
PCL/DEX-1	0.001	4.54 ± 0.20	0.72 ± 0.03
PCL/DEX-5	<0.001	4.50 ± 0.38	0.17 ± 0.01

**Table 3:** Viability of THP-1 cells after 24 h in vitro co-cultivation with PCL and PCL/DEX-1 scaffolds. Three-layer 3D-printed scaffolds were used for this assay. Data are presented as mean value ± SD (*n* ≥ 9).

Series	Cell Viability (%)	
	Without TNFα Treatment	With TNFα Treatment
Negative control	94.4 ± 0.2	94.1 ± 0.4
PCL	95.3 ± 0.4	90.8 ± 0.8 *^,^**
PCL/DEX-1	63.2 ± 1.9 *	57.9 ± 4.3 *

*—the difference with negative control (THP-1 cell without test specimens) were significant with *p* < 0.001; **—the difference between TNFα-untreated THP-1 cells in presence of scaffolds were significant with *p* < 0.001.

**Table 4:** CD54 expression by THP-1 cells without and with TNFα stimulation in vitro in presence of PCL and PCL/DEX-1 scaffolds. Three-layer 3D-printed scaffolds were used for this assay. Data are presented as mean value ± SD (*n* ≥ 9).

Series	CD54 Expression (MFI)	
	Without TNFα Treatment	With TNFα Treatment
Negative control	0.77 ± 0.09	5.40 ± 1.01
PCL	0.98 ± 0.10	2.30 ± 0.23 *
PCL/DEX-1	0.96 ± 0.04 *	1.35 ± 0.08 **

*, **—the difference with negative control (THP-1 cell without test specimens) were significant with *p* < 0.05 and *p* < 0.001.

## Data Availability

Data are available within the article and its Supplementary Materials.

## Supplementary Materials

The following supporting information can be downloaded at: https://www.mdpi.com/article/10.3390/polym15193957/s1, Figure S1: Compression stress-strain curves for 3D-printed (a) and monolithic (b) PCL specimens and its composites; Figure S2: Pore size distribution of different 3D-printed scaffolds (BET): (a) PCL; (b) PCL/CIP-1; (c) PCL/CIP-5; Figure S3: FTIR spectra of 3D-printed composite matrices with 5 wt % DEX (PCL/DEX-5) or CIP (PCL/CIP-5), as well as pristine PCL matrix and filler drugs; Table S1: Correlation coefficients and calculated parameters with different mathematical models of release; Figure S4: Regression curves obtained with different dissolution mathematical models of release; Table S2: Viability of THP-1 cells after 24 h in vitro co-cultivation with different concentrations of dexamethasone (DEX). Data are presented as mean ± SD (n ≥ 9); the data are shown as the percentages of viable cells; Table S3: CD54 expression by THP-1 cells in response to in vitro stimulation with different concentrations of dexamethasone. Data are presented as mean ± SD (n ≥ 9); the data are shown as CD54 MFI
