# Supplementary material for: Drug Loaded 3D-Printed Poly(ε-Caprolactone) Scaffolds for Local Antibacterial or Anti-Inflammatory Treatment in Bone Regeneration
Source: Polymers (Basel). 2023 Sep 30;15(19):3957. doi: 10.3390/polym15193957 (PMC10575412; doi:10.3390/polym15193957)
Supplement: Supplementary file 1 [file polymers-15-03957-s001.zip › polymers-2604194-supplementary.pdf]

*Supplementary Materials*

# Drug Loaded 3D-Printed Poly(ε-Caprolactone) Scaffolds for Local Antibacterial or Anti-Inflammatory Treatment in Bone Regeneration

**Mariia Stepanova [1], Ilia Averianov [1], Iosif Goffman [1], Natalia Shevchenko [1], Artem Rubinstein [2], Tatiana Egorova [3], Andrey Trulioff [2], Yulia Naschekina [4], Igor Kudryavtsev [2,5], Elena Demyanova [3], Evgenia Korzhikova-Vlakh [1] and Viktor Korzhikov-Vlakh [1,6]***

[1] Institute of Macromolecular Compounds, Russian Academy of Sciences, 199004 St. Petersburg, Russia; maristepanova@hq.macro.ru (M.S.), averianovilia@gmail.com (I.A.), gofman@imc.macro.ru (I.V.), natali.shevchenko29@gmail.com (N.S.), vlakh@hq.macro.ru (E.K.-V.)

[2] Institute of Experimental Medicine, 197376 St. Petersburg, Russia; arrubin6@mail.ru (A.R.), trulioff@gmail.com (A.T.)

[3] State Research Institute of Highly Pure Biopreparations FMBA of Russia, 197110, St. Petersburg, Russia; egorova25tat@yandex.ru (T.E.), lenna_22@mail.ru (E.D.)

[4] Institute of Cytology, Russian Academy of Sciences, St. Petersburg 194064, Russia; ulychka@mail.ru (Yu.N.)

[5] School of Biomedicine, Far Eastern Federal University, 10 Ajax Bay, Russky Island, 690922 Vladivostok, Russia; igorek1981@yandex.ru (I.K.)

[6] Institute of Chemistry, Saint-Petersburg State University, 198504 St. Petersburg, Russia;

* Correspondence: v.korzhikov-vlakh@spbu.ru

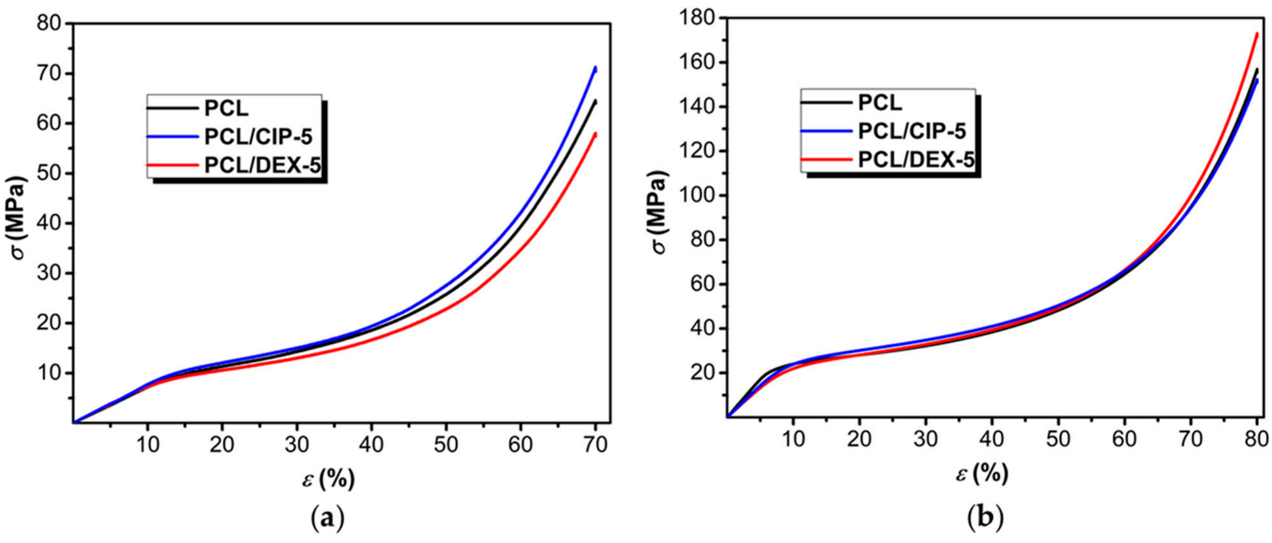


**Figure S1**. Compression stress-strain curves for 3D-printed (**a**) and monolithic (**b**) PCL specimens and its composites.

(a)

(b)

(c)

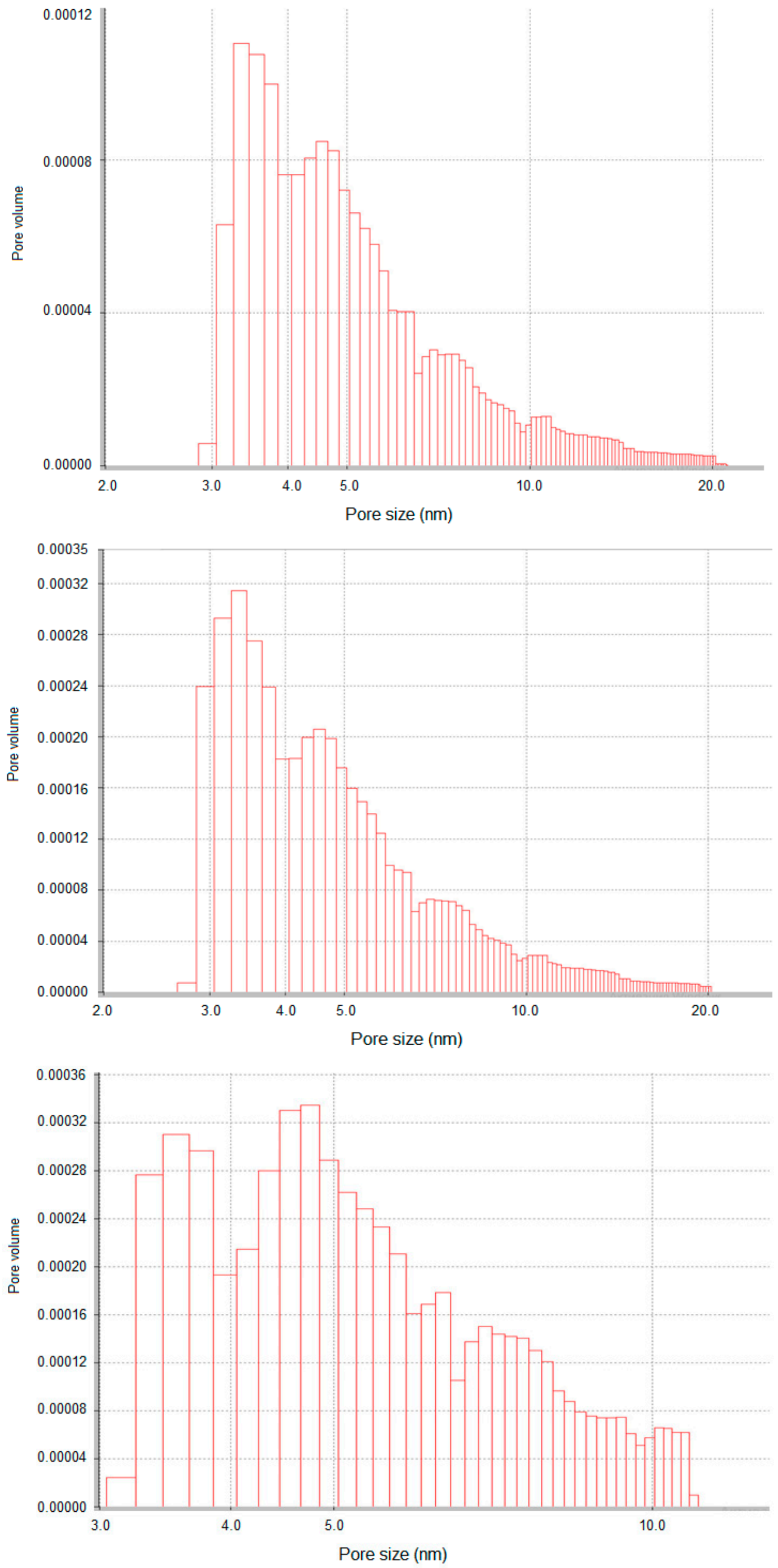


**Figure S2.** Pore size distribution of different 3D-printed scaffolds (BET): (**a**) PCL; (**b**) PCL/CIP-1; (**c**) PCL/CIP-5.

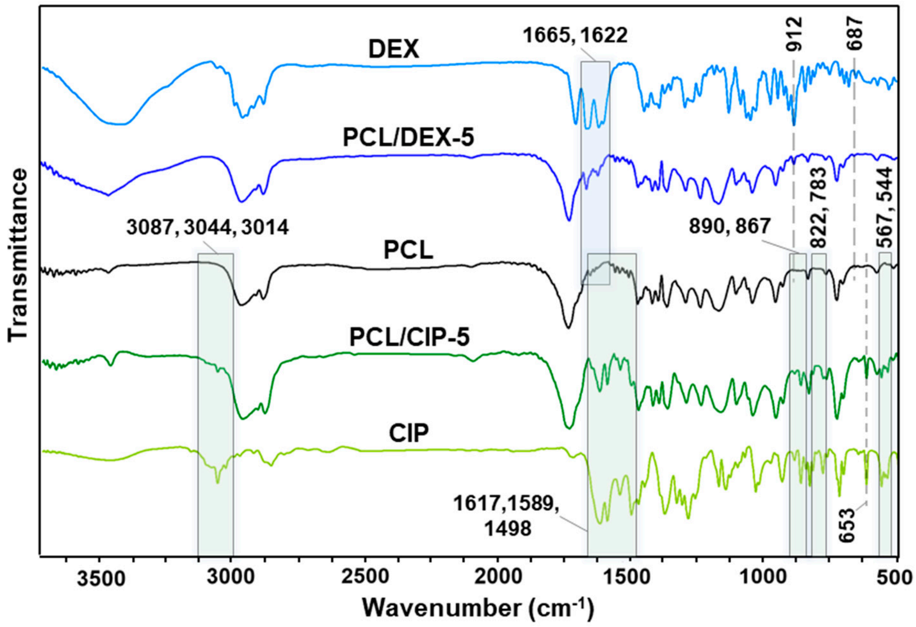


**Figure S3**. FTIR spectra of 3D-printed composite matrices with 5 wt% DEX (PCL/DEX-5) or CIP (PCL/CIP-5), as well as pristine PCL matrix and filler drugs.

**Table S1.** Correlation coefficients and calculated parameters with different mathematical models of release.

| **Model** | **Correlation coefficients and parameters** | **CIP** | | | | **DEX** | | | | | |
|---|---|---|---|---|---|---|---|---|---|---|---|
| | | **PB** | | **PB+Lipase** | | **PB** | | | **PB+Lipase** | | |
| | | **1 %** | **5 %** | **1 %** | **5 %** | **0.5 %** | **1 %** | **5 %** | **0.5 %** | **1 %** | **5 %** |
| **Zero-order** | $R^2$ | 0.9805 | 0.9802 | 0.9579 | 0.9779 | 0.8616 | 0.9071 | 0.9290 | 0.8747 | 0.9689 | 0.9879 |
| | $k_0$ | 1.62 | 1.10 | 2.23 | 1.15 | 0.23 | 1.91 | 0.83 | 0.26 | 2.56 | 0.96 |
| **First-order** | $R^2$ | **0.9905** | 0.9875 | 0.9761 | 0.9848 | 0.9055 | 0.9417 | 0.9345 | 0.9248 | **0.9917** | **0.9924** |
| | $k_1$ | 0.02 | 0.01 | 0.03 | 0.01 | 0.003 | 0.03 | 0.01 | 0.004 | 0.04 | 0.01 |
| **Higuchi** | $R^2$ | **0.9903** | **0.9915** | **0.9903** | **0.9962** | 0.9726 | 0.9819 | 0.9770 | 0.9776 | **0.9984** | 0.9880 |
| | $k_H$ | 7.48 | 5.06 | 10.67 | 5.41 | 3.20 | 9.35 | 4.03 | 3.70 | 12.18 | 4.46 |
| **Korsmeyer-Peppas** | $R^2$ | **0.9933** | **0.9948** | **0.9901** | **0.9978** | **0.9965** | 0.9896 | 0.9808 | **0.9971** | **0.9986** | **0.9963** |
| | $k_{KP}$ | 4.415 | 3.023 | 11.600 | 4.447 | 8.488 | 12.455 | 6.163 | 9.179 | 10.730 | 2.608 |
| | $n$ | 0.68 | 0.68 | 0.47 | 0.56 | 0.30 | 0.41 | 0.34 | 0.31 | 0.55 | 0.68 |
| **Hixon-Crowell** | $R^2$ | 0.9833 | 0.9827 | 0.9616 | 0.9743 | 0.8915 | 0.9312 | 0.9328 | 0.9095 | 0.9869 | **0.9911** |
| | $k_{HC}$ | $7.0\times10^{-3}$ | $4.0\times10^{-3}$ | $1.1\times10^{-2}$ | $5.0\times10^{-3}$ | $9.7\times10^{-4}$ | $8.0\times10^{-3}$ | $3.0\times10^{-3}$ | $1.2\times10^{-3}$ | $1.2\times10^{-2}$ | $4.0\times10^{-3}$ |
| **Hopfenberg** | $R^2$ | **0.9905** | 0.9875 | 0.9761 | 0.9848 | 0.8314 | 0.9416 | 0.9345 | **0.9406** | **0.9916** | **0.9923** |
| | $k_{HB}$ | $3.9\times10^{-5}$ | $1.0\times10^{-5}$ | $1.7\times10^{-5}$ | $1.2\times10^{-5}$ | $7.1\times10^{-7}$ | $3.2\times10^{-5}$ | $3.3\times10^{-5}$ | $5.6\times10^{-7}$ | $3.2\times10^{-5}$ | $1.0\times10^{-4}$ |
| **Weibull** | $R^2$ | **0.9972** | **0.9980** | **0.9908** | **0.9976** | **0.9986** | **0.9907** | 0.9799 | **0.9955** | **0.9975** | **0.9974** |
| | α | 19.484 | 26.822 | 8.989 | 24.183 | 5.101 | 7.630 | 17.555 | 15.263 | 10.707 | 43.009 |
| | β | 0.71 | 0.66 | 0.59 | 0.64 | 0.19 | 0.47 | 0.41 | 0.45 | 0.72 | 0.76 |

| | | | | | | | | | | | |
|---|---|---|---|---|---|---|---|---|---|---|---|
| **Makoid-** | $R^2$ | **0.9963** | **0.9972** | **0.9942** | **0.9985** | **0.9990** | **0.9920** | **0.9902** | **0.9968** | **0.9989** | **0.9976** |
| **Banakar** | $k_{MB}$ | 3.946 | 2.681 | 12.493 | 4.571 | 6.346 | 11.413 | 6.866 | 9.982 | 10.898 | 2.596 |
| | $n$ | 0.776 | 0.785 | 0.397 | 0.526 | 0.392 | 0.505 | 0.187 | 0.296 | 0.541 | 0.672 |
| | $k$ | 0.008 | 0.009 | 0.007 | 0.004 | 0.001 | 0.010 | 0.017 | $1.1\times10^{-4}$ | $3.6\times10^{-4}$ | 0.002 |
| **Peppas-** | $R^2$ | **0.9975** | **0.9981** | **0.9940** | **0.9983** | **0.9991** | **0.9918** | 0.9826 | **0.9967** | **0.9989** | **0.9976** |
| **Sahlin** | $k_1$ | 15.61 | 10.37 | 9.76 | 4.32 | 5.97 | 11.85 | 4.60 | 9.41 | 10.97 | 0.55 |
| | $k_2$ | 18.10 | 12.19 | 2.32 | 0.18 | 0.19 | 0.71 | 1.15 | 0.14 | 0.01 | 2.07 |
| | $m$ | 0.21 | 0.21 | 0.35 | 0.52 | 0.45 | 0.55 | 0.29 | 0.30 | 0.54 | 0.37 |

**Zero-order** $F=k_0*t$

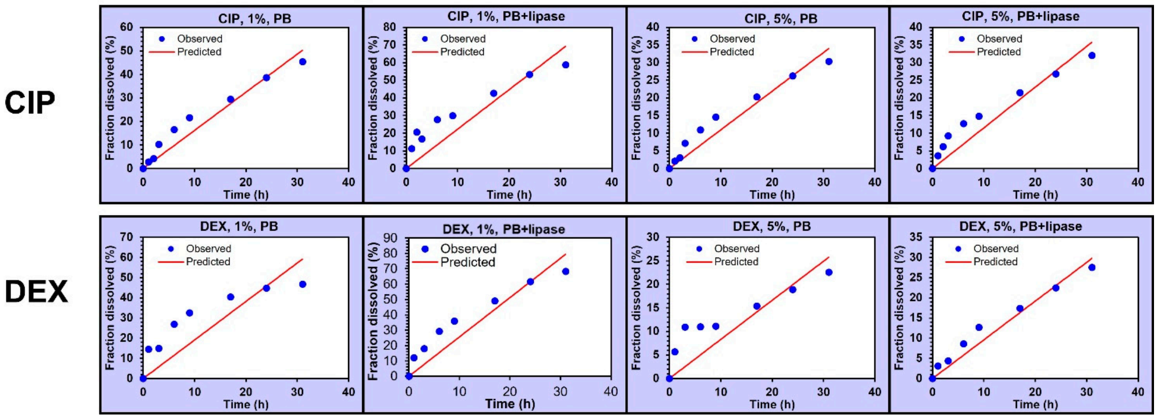


**First-order** $F=100*[1-Exp(-k_1*t)]$

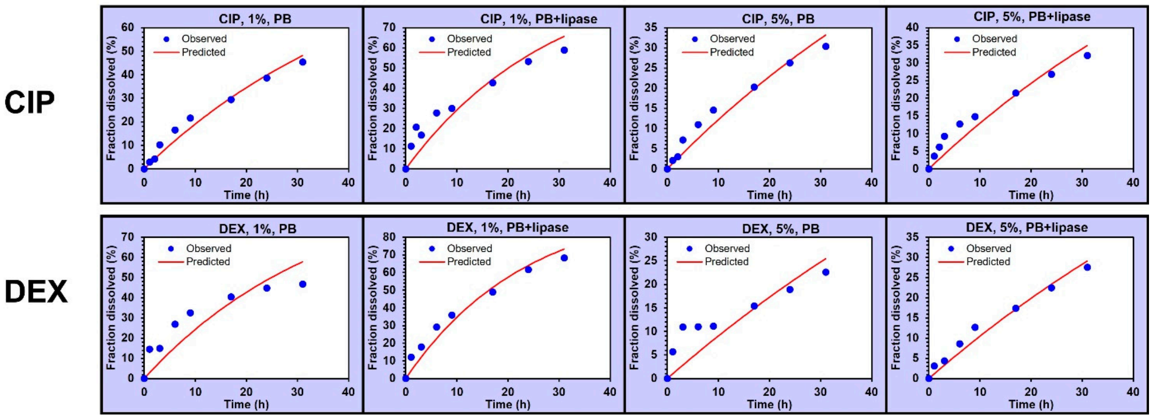

## Higuchi $F=k_H*t^{0.5}$

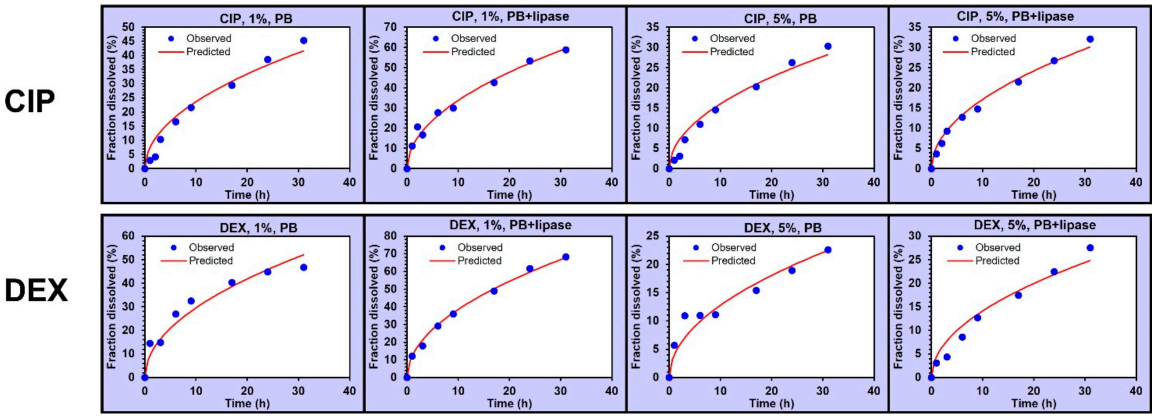


## Korsmeyer-Peppas $F=k_{KP}*t^n$

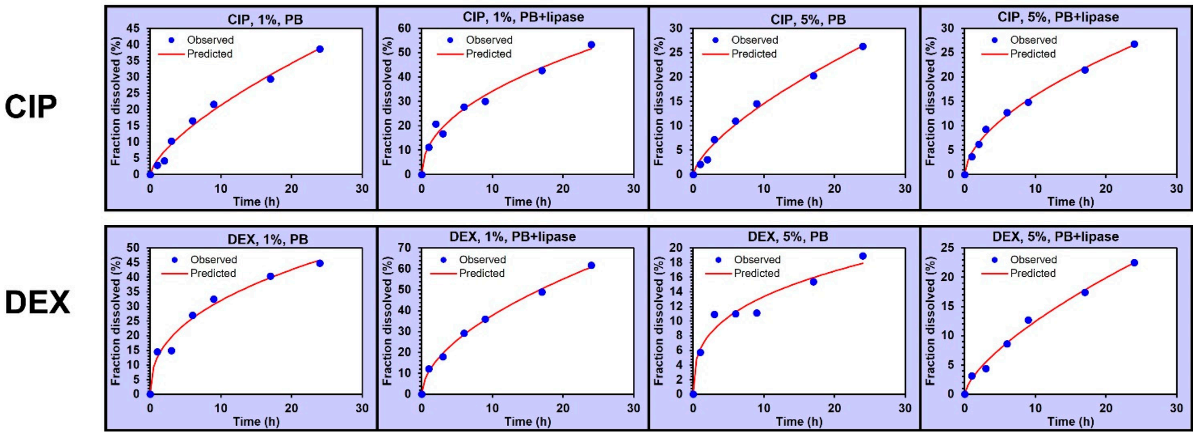


## Hixon-Crowell $F=100*[1-(1-k_{HC}*t)^3]$

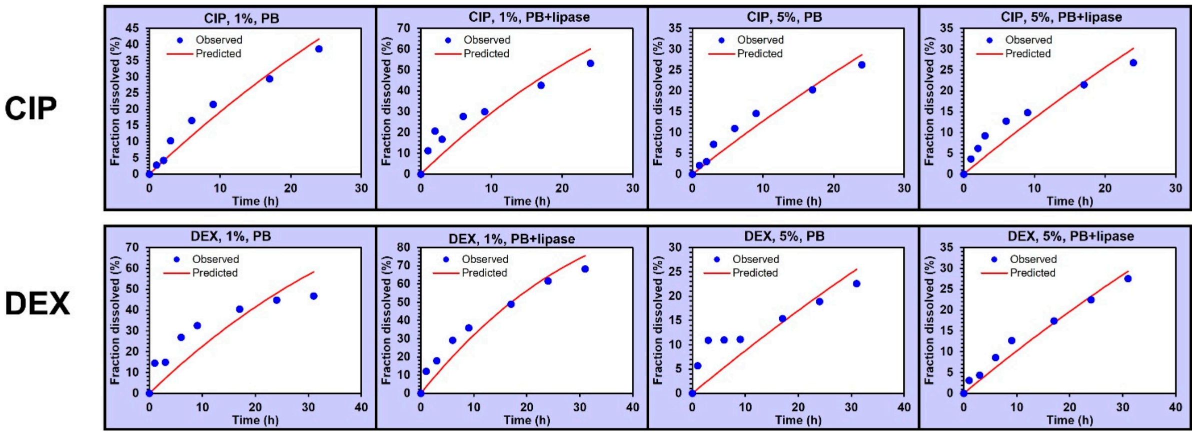

## Hopfenberg

$$F=100*[1-(1-k_{HB}*t)^n]$$

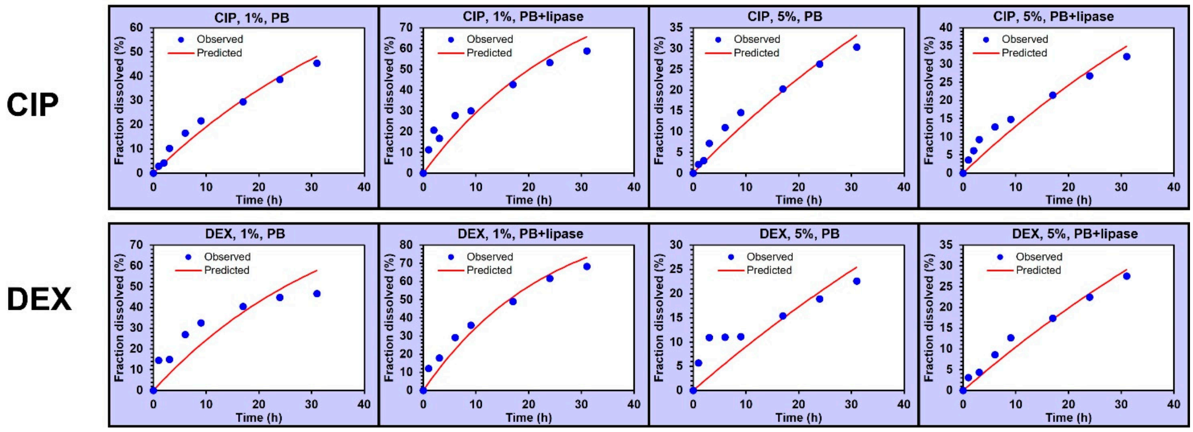


## Weibull

$$F=100*\{1-Exp[-((t-Ti)^{\beta)/\alpha}]\}$$

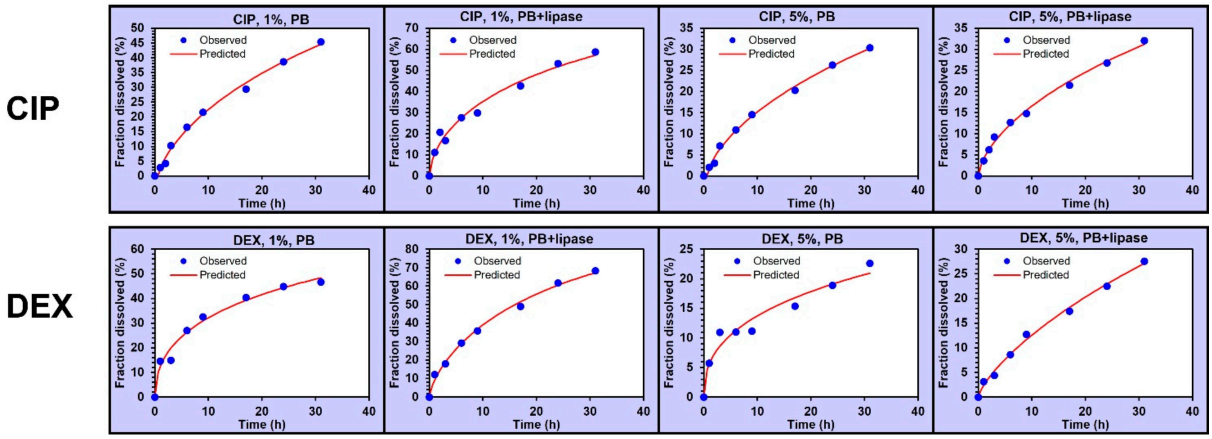


## Makoid-Banakar

$$F=k_{MB}*t^n*Exp(-k*t)$$

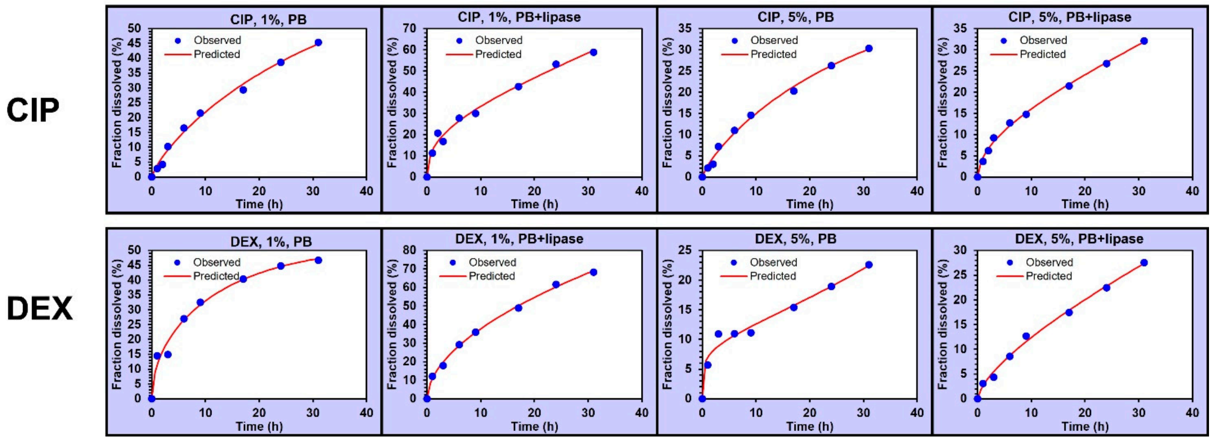

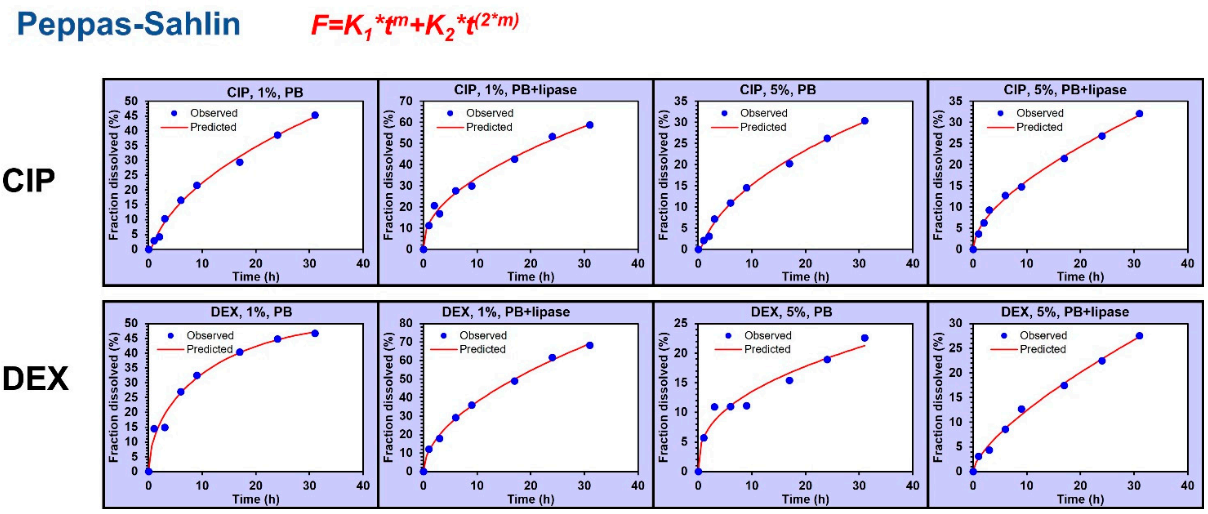


**Figure S4**. Regression curves obtained with different dissolution mathematical models of release.

**Table S2**. Viability of THP-1 cells after 24 h *in vitro* co-cultivation with different concentrations of dexamethasone (DEX). Data are presented as mean ± SD (n ≥ 9); the data are shown as the percentages of viable cells.

| | Cell viability (%) | | | | | |
|---|---|---|---|---|---|---|
| | without TNFα treatment | | | with TNFα treatment | | |
| **Series** | **Viable** | **early Ap** | **late Ap/Necrosis** | **Viable** | **early Ap** | **late Ap/Necrosis** |
| Negative control | 94.43 ± 0.21 | 4.36 ± 0.24 | 1.06 ± 0.07 | 94.09 ± 0.41 | 4.10 ± 0.37 | 1.61 ± 0.12 |
| DEX, 5 µg/mL | 94.82 ± 0.20 | 4.19 ± 0.23 | 0.87 ± 0.06 | 92.16 ± 0.27 ** | 5.47 ± 0.22 * | 2.12 ± 0.12 * |
| DEX, 10 µg/mL | 94.75 ± 0.25 | 3.77 ± 0.29 | 1.07 ± 0.09 | 90.06 ± 1.12 *** | 6.86 ± 0.74 ** | 2.70 ± 0.41 |
| DEX, 20 µg/mL | 93.81 ± 0.26 | 4.55 ± 0.20 | 1.41 ± 0.14* | 83.13 ± 1.28 *** | 11.71 ± 1.08 *** | 4.64 ± 0.45 *** |
| DEX, 30 µg/mL | 93.60 ± 0.33 | 4.49 ± 0.30 | 1.63 ± 0.12 *** | 83.51 ± 1.39 *** | 11.49 ± 0.98 *** | 4.50 ± 0.52 *** |
| DEX, 50 µg/mL | 94.96 ± 0.31 | 3.13 ± 0.22 ** | 1.90 ± 0.21 ** | 85.34 ± 1.08 *** | 11.45 ± 1.00 *** | 3.021 ± 0.12 *** |
| DEX, 100 µg/mL | 86.73 ± 0.87 *** | 11.46 ± 0.81 *** | 1.81 ± 0.07 ** | 59.41 ± 1.79 *** | 33.78 ± 1.69 *** | 6.81 ± 0.19 *** |

*, **, *** – the difference with negative control (THP-1 cell without dexamethasone) were significant with $p < 0.05$, $p < 0.01$ and $p < 0.001$ according to non-parametrical Mann–Whitney U test; ‘Viable’ – YO-PRO-1–PI– cells; ‘early Ap’ – THP-1 cell in early steps of apoptosis with YO-PRO-1+PI– phenotype; ‘late Ap/Necrosis’ – dead THP-1 cells with YO-PRO-1+PI+ phenotype.

**Table S3**. CD54 expression by THP-1 cells in response to *in vitro* stimulation with different concentrations of dexamethasone. Data are presented as mean ± SD (n ≥ 9); the data are shown as CD54 MFI.

| Series | CD54 expression (MFI) | |
|---|---|---|
| | **w/o TNFα treatment** | **with TNFα treatment** |
| Negative control | 0.773 ± 0.084 | 5,397 ± 1,008 |
| DEX, 5 μg/mL | 0.718 ± 0.027 | 2.489 ± 0.489 ** |
| DEX, 10 μg/mL | 0.744 ± 0.031 | 2.083 ± 0.253 * |
| DEX, 20 μg/mL | 0.855 ± 0,043 | 2.237 ± 0.097 * |
| DEX, 25 μg/mL | 1.022 ± 0.068 * | 2.398 ± 0.218 * |
| DEX, 30 μg/mL | 0.852 ± 0.066 | 1.998 ± 0.075 ** |
| DEX, 50 μg/mL | 0.792 ± 0.029 | 1.696 ± 0.082 *** |
| DEX, 100 μg/mL | 0.852 ± 0.033 | 1.403 ± 0.085 *** |

*, ** – the difference with negative control (THP-1 cell without dexamethasone) were significant with $p < 0.05$ and $p < 0.01$ according to non-parametrical Mann–Whitney U test.
